# Evaluating the potential of suburban and rural areas for tourism and recreation, including individual short-term tourism under pandemic conditions

**DOI:** 10.1038/s41598-022-24503-z

**Published:** 2022-11-27

**Authors:** Anna Bielska, Andrzej Szymon Borkowski, Adrianna Czarnecka, Maciej Delnicki, Jolanta Kwiatkowska-Malina, Monika Piotrkowska

**Affiliations:** Faculty of Geodesy and Cartography, Warsaw University of Technology, Politechniki 1 sq., 00-661 Warsaw, Poland

**Keywords:** Environmental sciences, Computational science

## Abstract

Limited mobility and restrictions in social life caused by the COVID-19 pandemic changed people’s recreational behavior and made them seek more contact with nature. As a result, the provision of new recreational spaces in the vicinity of cities gained importance. In conditions of social distancing, rural and suburban areas can be an attractive alternative to individual short-term tourism, satisfying the need for recreation and mental and physical health restoration of urban residents. In the study a methodology for assessing the tourist and recreational potential of the area (METPRET) concerning the recreational behaviors identified in the pandemic was proposed. It includes the Recreational Potential Index (RPI), which comprises four criteria: landscape values and socio-economic conditions; environmental protection; air quality; transportation accessibility. The application of the methodology is exemplified in the Mazovia Voivodeship, Poland. The research allows the determination of characteristics that potential recreation areas should have under pandemic conditions. The RPI makes it possible to select new rural and suburban areas attractive for short-term tourism. Designating additional recreational areas may contribute to the dispersion of users in existing green areas in cities, which is particularly important during a pandemic.

## Introduction

Recreational activities bring health benefits to humans, stimulate social interactions and ensure efficient use of time. The COVID-19 pandemic has affected the health of the population both via the direct effects of the disease and also via stress caused by fear of infection, the need to change current ways of life and work, loss of income, limitation of social contacts, family tensions and limited opportunities to solve a challenging situation. In the face of such conditions, the health function of tourism has gained a new meaning, understood not only as regeneration after illness but also as restoring energy used up in everyday work and eliminating fatigue caused by the negative consequences of modern civilization^1^.

Before 2020, studies on the impact of pandemics on the tourism and leisure sector were conducted only in countries where epidemics had previously occurred (e.g. the Severe Acute Respiratory Syndrome (SARS) epidemic in 2003 in Far East countries, the Middle East Respiratory Syndrome (MERS) epidemic in 2012 in South Korea and Ebola virus in 2014 in West Africa). They focused primarily on economic analysis and changing recreational behavior was not of broad interest to researchers until the outbreak of the COVID-19 pandemic^2^. This issue should be considered significant when considering the need to limit the effects of the pandemic on the tourism economy. An analysis of tourist behavior during a pandemic is needed to plan current recovery actions, as well as to develop solutions to influence tourist demand in new conditions that will be possible to use in potential future crises^2–8^.

As a result of the COVID-19 pandemic, the tourism industry saw a greater interest in travel to destinations closer to home, including by car, which resulted from the need to maintain a sense of security^3,9–12^. Studies conducted in Poland by Zajadacz et al.^7^ showed that during the pandemic in 2020, around 70% of respondents spent holidays away from home, mainly in the country (78%). Two destinations were noted: well-known tourist resorts by the sea or in the mountains, and less popular regions, including own recreational plots and family homes in the countryside^7,11,13^. Trips were organized primarily independently, with people eschewing travel agencies or other organizations out of fear of planning too many weeks ahead^7,10^. According to press reports, these observations were also valid in the context of the 2021 holiday season^9,14–16^. Central Statistical Office data show that in July 2021, the number of domestic tourists was 33.2% higher than in July 2020 and 1.9% higher than in 2019^17^.

The most common motivation when respondents were asked about the holiday season in 2020 was to take trips to the broadly understood ‘womb of nature’^3,7^. Similar observations are provided by analyzing trends from the Google search engine; when looking for vacation ideas, Poles were more likely to choose accommodation located ‘close to nature’^16^. This explains the increase in popularity of agritourism and camping^3,6,11^, as well as the emergence of a new phenomenon of glamping (glamorous camping—a combination of camping with luxury)^16^. The pandemic has influenced the development of new leisure preferences, including trends combining leisure with work and business, such as ‘workation’ or ‘bleisure’. This has helped the emergence of new holiday and weekend destinations away from crowded resorts^16^.

As a result of the pandemic, in connection with limited mobility and restrictions in social life, the search for opportunities to engage in tourist and recreational behaviors in open spaces, particularly in the vicinity of large urban centers, has gained a new meaning. In this context, it is expected that short-term tourism in rural areas will gain momentum^12,18,19^. Even before the COVID-19 pandemic, Bański^20^ indicated the potential for the development of weekend recreation in rural areas of Poland; this is a common way of spending free time for approximately 25% of city dwellers in the European Union, but only for 8% in Poland. Concerning the Mazovia region, the need to redirect tourist traffic outside the borders of Warsaw, the dominant center in the region, was also emphasized in the Report on Research on Tourist Traffic in the Mazovia Voivodeship in 2015–2017^21^. The report highlighted the need to solve the disparity between the voivodeship’s center and periphery and proposed that the tourist offer should be developed to meet the weekend relaxation needs of the inhabitants of Warsaw and other large cities in the region.

The COVID-19 pandemic has led to a great need for convalescence and rebuilding of physical and mental health in the open air, in contact with nature^22^. It is necessary to find new areas for individual short-term tourism in conditions of social distancing. Designating additional recreational areas will contribute to relieving those within the administrative boundaries of cities.

The present research aimed to identify rural and suburban areas that may be allocated for tourist and recreational functions. We sought to answer the following research questions:

- What features should be characteristic of potential areas for tourist and recreational purposes in a pandemic?
- What methodology for identifying areas for tourist and recreational functions should be used to prepare planning documents in rural and suburban areas?

## Materials and methods

In conditions of limited social mobility, rural and suburban areas may relieve pressure on urban recreational areas, as their tourist potential has not been fully used. Such possibility is significant in pandemic conditions when people are looking for areas where they will be able to engage in active recreation while being highly dispersed. Moreover, rural areas are usually free from air pollution, and can therefore support the healthcare sector in both the restoration of physical and mental health.

The thematic scope of the present article included identification of tourist and recreational behaviors during the COVID-19 pandemic, and determination of the method for identifying potential areas that could meet the needs of city residents in terms of individual short-term tourism (understood as trips lasting from a few hours to two-to-four days, tailored to individual needs, targeted thematically^23^. The presented issues were investigated using deductive reasoning.

This aim required the development of a methodology for assessing the tourist and recreational attractiveness of the area, and then testing this methodology on the example of the Mazovia Voivodeship. The research indicates the types of data and data sources needed to determine the tourist and recreational potential of areas that have previously fulfilled other functions. The study was conducted in three stages:

- Review of the literature in search of (a) best practices for the organization of individual short-term tourism in conditions of limited social mobility and (b) methods of assessing the potential of rural and suburban areas to fulfil tourist and recreational functions. Literature studies, including scientific and grey literature, statistics, poll results and reports, were examined to determine the types of recreational behavior during a pandemic. Factors influencing the attractiveness of areas in conditions of limited social mobility were also determined.
- Development of a methodology for determining and assessing the tourist and recreational potential of rural and suburban areas, including for individual short-term tourism, in pandemic conditions. Based on the literature and the results of analyses of environmental, communication, demographic, and socio-economic conditions in the Mazovia Voivodeship, criteria were selected and parameters important for assessing the potential of rural and suburban areas were established. Next, the types of data and sources relevant for determining and assessing tourist and recreational potential were selected and used to develop the Recreational Potential Index (RPI). The adopted indicators are constant and can be implemented in areas with different environmental, communication, demographic, and socio-economic conditions. However, the selection of parameters depends on the specificity of the area to be assessed. The developed methodology for identifying potential areas for tourist and recreational functions (METPRET) using the RPI is presented in a schema in Supplementary Figure S1.
- Testing of the developed methodology in the Mazovia Voivodeship, using quantitative and qualitative methods and spatial analyses in rural and urban–rural communes. The qualitative assessment included determining the criteria of the suitability of areas for tourist and recreational purposes, considering environmental, communication, demographic and socio-economic conditions and recreational behaviors identified in pandemic conditions. The quantitative assessment quantifies the potential for developing the tourist and recreational functions in conditions of limited mobility by determining the RPI.

The following data sources were used:

- the National Register of Boundaries (NRB), covering the borders of communes in the Mazovia Voivodeship
- OpenStreetMap and Topographic Objects Database, for the communication network and land cover
- the Land and Building Register (LBR), for the area and types of land
- the Agency for Restructuring and Modernization of Agriculture (ARMA), for the agrarian structure of farms
- the Central Statistical Office (Statistics Poland), characterizing the population and economic situation of the communes
- the General Directorate for Environmental Protection (GDEP), for forms of nature protection
- Air quality data from the Airly measurement stations database.

The study used the basic and advanced functionalities of Esri’s ArcGIS Desktop software package, which allowed for collecting, analyzing, and processing spatial data.

## Best practices related to organizing recreation in conditions of limited social mobility

The COVID-19 pandemic changed the recreational behavior of the population in terms of types of activities, frequency of their taking up, and places chosen for recreation, which was caused by restrictions on the mobility and access to the existing recreational areas.

### The need for contact with nature

Numerous publications highlight increased public demand for outdoor recreation in response to the pandemic^24–29^. However, as noted by Morse et al.^30^, the motivations underlying human interaction with nature in this situation are not sufficiently analyzed. Samuelsson et al.^28^ emphasize that understanding changes in recreational behavior is essential for the management and planning process of green areas to provide access to ecosystem services related to recreation.

Survey results show that among the ecosystem services provided by green areas, the most valued were cultural services, including positive impact on mental health and the chance to enjoy the beauty of nature, smells and sounds, and maintaining physical fitness through outdoor activity^30,31^. Importance was also attached to spiritual experiences during closeness to nature and its role in shaping individual identity^30^. Being surrounded by nature has thus become a way of dealing with an emotional crisis^24,28,32^. These results are confirmed by numerous studies on the soothing effect of nature on the functioning of the human brain, which manifests itself in increased sense of happiness, health and cognitive abilities^33,34^.

### Recreational behavior during the pandemic

Surveys conducted in the United States and Canada^26,30,31,35^ show that during the pandemic, interest in activities such as walking, jogging, gardening and cycling increased the most. In addition, much more attention was paid to outdoor activities that did not require physical effort, such as bird and nature watching, photography, relaxing in a hammock, playing with pets, or collecting plants and mushrooms. Climbing, backpacking, or skiing was practiced less frequently (see Supplementary Table S1). The less difficult activities have grown in popularity as they do not require specialized equipment or high skill levels and can be practiced almost anywhere without the need for long trips or the involvement of other participants^30^. In addition, as recommended by governments, people were advised to avoid activities associated with increased risk of trauma or occurring in remote environments, such as rock climbing or water sports, to avoid additional strain on health systems^27^.

Among the recreational activities of Poles in the first phase of the pandemic (March–May 2020), there was a noticeable increase in passive activities (watching television, listening to music, playing computer games, and reading books). It was not until the summer period (June–August 2020) that there was an increased interest in outdoor activities such as walking, cycling, kayaking, and hiking. Gardening on recreational plots and farmland, performed with family in the countryside, was also popular^7^. The survey conducted by Cybulska and Pankowski^36^ provided similar results, where 20% of respondents expressed a positive opinion about the more frequent cultivation of plants on their balconies or in the garden in the aftermath of the pandemic. However, a significant proportion of respondents report a high probability of returning to their old recreational habits when the threat from the virus is considered minimal^31,37^.

### Recreational behavior – travel distance change

For years, suburban areas have been a place of recreation and relaxation for residents of large cities. Increased accessibility from public and individual transport contributed to a substantial expansion into open suburban areas^38^. Recreation areas located approximately 20–25 km away from cities with a population of half a million to one million people meet the needs of daily long-distance and weekly short-distance relaxation^39,40^. Transport accessibility of recreational areas is also often defined as within 30 km, corresponding to 60 min, as the maximum time spent on one-day trips^41^.

The pandemic has seen further increases in suburban tourism. During the lockdown in 2020, Parisian traffic was limited to short journeys (up to 29 km) from the city limits, compared to pre-pandemic, where 95% of traffic extended to a radius of 201 km^42^. In the United States, nearly 90% of recreational behaviors during the initial phase of the pandemic were concentrated within 15 miles (about 24 km) of place of residence, whereas previously, nearly three-quarters of such activities occurred more than 15 miles away, with most destinations between 24 and 80 km^35^. Similarly, in Canada, about 70% of the population chose places within 30 min during the pandemic, which is a 10% increase on the pre-pandemic period^31^. Thus, areas close to place of residence, such as a housing estate or even one’s own flat/house, were chosen more often for recreation^26,43^. Finally, there has been an increase in pedestrian activity in city parks, suburban forests and protected areas, which underlines the importance of access to open green spaces close to the built-up mass of cities^29^.

## Tourist and recreational attractiveness of suburban and rural areas in the conditions of a pandemic

Tourist attractiveness is a complex phenomenon determined by tourist values, existing tourist infrastructure, transport accessibility^44^ and the state of the natural environment^45^.

### Tourist values

Tourist values are specific features of the natural environment and the results of human activities which are of interest to tourists^44^. Motives for tourist activity can include recreational values for the regeneration of physical and mental health, sightseeing values for cognitive interest and specialized values for qualified tourism and spa treatment^46^. However, attractiveness is also a subjective feeling and aspects such as silence, peace, the hospitality of residents, new acquaintances or knowledge about the region can attract tourists^47^.

When assessing tourist attractiveness, features of the natural environment such as topography, surface water, flora and (to a lesser extent) cultural values are most often considered. Attention is also paid to the presence of nuisances, such as transit lines or industrial and storage zones, which may reduce scenic values and cause environmental pollution, discouraging potential users^48,49^. A summary of criteria and indicators used in attractiveness assessments is presented in Supplementary Table S2.

First, criteria concerning the type of land cover, topography, landscape diversity, the presence of unique natural or man-made elements (e.g. monuments, museums, places of worship) and legal forms of nature protection are considered^48,50–59^. They determine the landscape’s scenic, ecological, and cultural values and its suitability for specific forms of recreation. For example, areas covered by legal protection reflect exceptional high-value areas perceived by tourists as particularly worth visiting^5,60–64^. Such areas often become attractive for recreation, which was demonstrated in particular by numerous examples during the Covid-19 pandemic^5,65,66^.

Among the criteria, there is also the state of the natural environment defined in relation to water purity^50,53^, air quality and noise level^48,53,58,67^ or the amount of recovered waste^53^. The inclusion of information on the quality of the components of the natural environment, including air, surface water and the acoustic climate, should be considered necessary as they directly affect comfort and health^58^.

Another vital aspect is climatic conditions. During the assessment for recreation purposes, knowledge about the climate as well as the body’s reaction to various atmospheric situations (e.g. the body’s thermal economy) is required to make optimal use of climatic resources^68^. For outdoor recreation, the stability of weather conditions is of great importance to maintain full psycho-physical performance and eliminate the need for the human body to adapt^69^. Bioclimatic indicators relating to meteorological or biometeorological data averaged over many years are used to compare conditions on a regional or global scale^68,70,71^. Among the factors considered are, e.g. atmospheric pressure, sunshine duration, cloudiness, cooling power, effective-radiative temperature, long-lasting precipitations, and fogs^59,68,72–74^.

Finally, the accessibility of the area, understood not only as the transport accessibility of individual attractions or tourist service facilities, but also price, time and information availability^21,75^ should be considered. Moreover, a significant role in the assessment may have the level of development of recreational infrastructure including accommodation, catering and facilities like for example golf courses, tennis courts or hiking trails^58,59,75–77^ (see Supplementary Table S2).

### Correlation between air quality and COVID-19

There is great interest in determining how the unprecedented restrictions during the pandemic affected air quality, as the related findings have significant implications for public health and environmental policy^78^. One of the substantial and immediate effects of lockdown was a significant global reduction in air pollution, especially in the most industrialized countries^79^. Research from 27 countries shows that NO_2_ concentration decreased by 13–44%, O_3_ by 2–20% and PM2.5 (fine particles < 2.5 μm in diameter) by 10–28%^29^. Data from the Sentinel-5P satellite of the European Space Agency (ESA) confirm that at the turn of January and February 2020, the level of NO_2_, produced mainly by road transport, was reduced by up to 40% compared to the same period in 2019. Additionally, in the initial 14 days of lockdown, PM2.5 concentrations fell by 9%^29^.

Research on CO_2_ emissions has been conducted in 69 countries, 50 American states and 30 Chinese provinces, where 85% of the Earth’s population lives and 97% of global CO_2_ emissions are produced. It shows that at the beginning of April 2020, there was a 17% average reduction in global CO_2_ emissions compared to the same period in 2019 and in some countries, up to 26%. Keeping the restrictions for the following years would reduce global CO_2_ emissions by up to 7% annually^80^. Thus, paradoxically, the limitations caused by a pandemic may result, in particular, in reducing air pollution and improving human health^81^.

The SARS-CoV-2 coronavirus, which causes the COVID-19 disease, is easily transmitted by droplets when coughing, talking or sneezing. Mortality from COVID-19 depends on comorbidities, including conditions that increase cardiovascular risks such as high blood pressure, diabetes, obesity, coronary artery disease, as well as respiratory diseases such as asthma and chronic obstructive pulmonary disease, similar to those affected by air pollution^82^. The first reports suggesting that air pollution could be considered a silent ally for COVID-19 have appeared since the March 2020 lockdown. Poor air quality, especially high content of PM2.5 (the main component of smog), is one of the main risk factors and causes many excessive deaths^83,84^. According to estimates^85^, about seven million people die each year from excessive air pollution, and 91% of the population lives in areas where air pollution exceeds permissible levels. Long-term exposure to PM2.5 and O_3_ dust causes the death of 8.8 million people annually^82^. Thus, smog may facilitate the transmission of the SARS-CoV-2 coronavirus and increase disease incidence. Compounds found in smog damage the epithelium of the respiratory tract (the first barrier between the air and internal organs), opening the door to various types of pathogens, including coronavirus. In addition, the virus is transmitted through dust, which means it stays in the air longer.

In December 2020, the results of a study were published comparing satellite data on air pollution with PM2.5 dust in the course of two epidemics: SARS in 2003 and the current COVID-19 pandemic^86^. There was a strong correlation between air quality and the number of COVID-19 cases. Up to 15% of deaths can be associated with the concentration of PM2.5 in the air. In Asia, the most polluted region globally, smog may be complicit in up to 27% of deaths, in Europe, 19% and in the United States, 17%. It is estimated that if it were not for the effects of smog, the death rate from COVID-19 in Poland would have been 28% lower. A very high level of air pollution increases with each heating season and overlaps with the new waves of disease. In Poland, most buildings do not have mechanical ventilation with air filters. As a result, the air entering buildings is almost as polluted as the air outside and there is a constant high risk of inhaling PM2.5 particles. The flu season and increased infections of the upper respiratory tract coincide with the heating season when the concentration of PM2.5 is very high.

Scientists at the Harvard T.H. Chan School of Public Health in Boston indicated that the risk of dying from coronavirus is increased in people who live in areas with high air pollution^87^. They found that an increased concentration of PM2.5 is associated with an 8% increase in COVID-19 mortality rate. This means that a slight increase in long-term exposure to PM2.5 increases the rate of COVID-19 deaths. The obtained data were adjusted by 20 confounding factors, including population size, age, population density, time since a stay-at-home order, number of hospital beds and people surveyed, and socio-economic variables (e.g. obesity and smoking). For total reliability, as many as 80 sensitivity analyses were carried out, but the data obtained in the study remained resistant to secondary and sensitivity analyses.

Mortality dropped significantly in Europe and the United States during the second wave of COVID-19^88^. The Netherlands recorded the most significant decrease in mortality (16.17), followed by Denmark (14.28), France (13.67), Belgium (11.25) and other Western European countries, all of which were greater than the countries of Eastern Europe and the United States. There is a certain structural similarity between Europe and the United States, where the north-eastern states were most successful. According to the authors, this may be due to several facts: underestimation of the number of cases during the first wave, the fact that deaths in the first wave disproportionately affected the elderly, and the fact that during the second wave, the infection mainly affected younger people. Lower mortality rates were primarily seen in countries with widely accessible health care.

The analysis of the COVID-19 death rates in areas with significant air pollution and the current research indicates a strong correlation between poor air quality and increased mortality, infectivity or more severe disease. Even short-term exposure to air pollutants can reduce the resistance of the respiratory tract to viruses and bacteria. Many of those who have recovered from COVID-19 still struggle with complications that involve the respiratory system, heart and other organs. There are concerns that long-term and short-term exposure to air pollution will see a worsening of many patients. In the case of high concentrations of pollutants in the air in hospitals, an increased number of admissions is generally recorded due to respiratory and heart conditions. Research is needed to distinguish the impact of air quality from other factors, such as population density. Research and statistical analysis should continue based on the increasing amount of available data.

## Method of assessing tourist and recreational potential of rural and suburban areas of the Mazovia Voivodeship for individual short-term tourism in pandemic conditions.

### Characteristics of the research area

**Figure 1:**
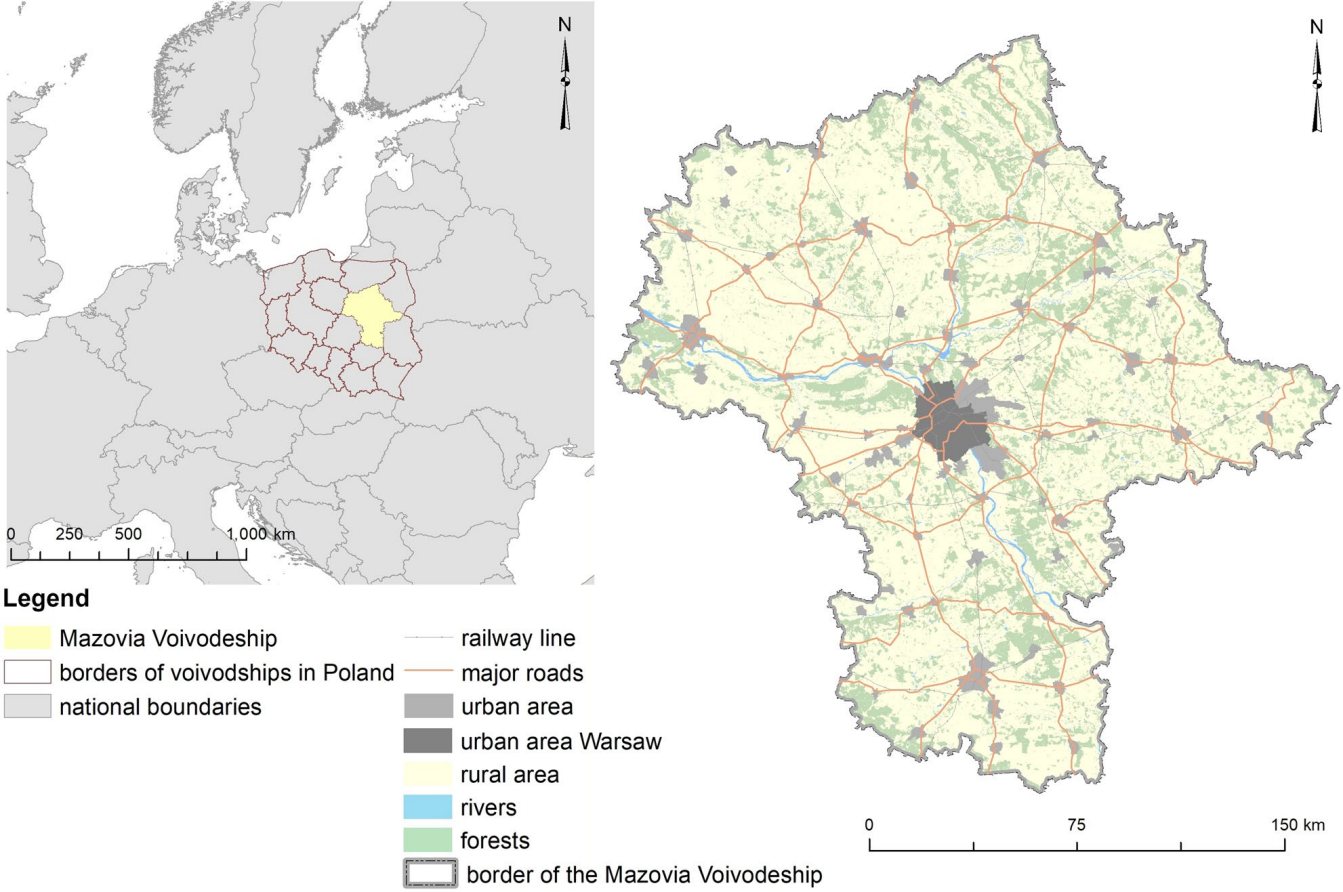
Location of the research area in Poland and Europe (Source: Own elaboration in ESRI ArcMap 10.8 based on Topographic Objects Database and NRB).

Rural and urban–rural communes in the Mazovia Voivodeship were selected as the research area (see Fig. 1). The Mazovia Voivodeship is in the central-eastern part of Poland and is the largest voivodeship in terms of area (over 35,558 km^2^) and population (over five million inhabitants).

The capital of the Mazovia Voivodeship (and the capital of Poland) located in Warsaw is inhabited by approximately 1.7 million people. Around two-thirds (64.4%) of the voivodeship’s population live in cities. The average population density is 153 people per km^2^ (in Poland overall it is 123 people per km^2^). Mazovia is subdivided into 37 powiats and five cities with powiat rights, the former being further divided into 314 communes, among which there are 35 urban, 50 urban–rural and 229 rural communes. It is one of the four fastest growing voivodeship in terms of population and this trend will continue until 2028^89^. The highest population growth is noticeable in powiats located near Warsaw due to the economic migration. A visible tendency is also migration of inhabitants to places away from the center, which contributes to the progressive suburbanization. The Mazovia Voivodeship shows spatial variability in terms of economic development between agglomerations (mainly Warsaw) and its agricultural periphery^90^.

Mazovia Voivodeship covers 13.4% of arable land in Poland, but this area is decreasing due to allocating it for non-agricultural purposes, including for housing^89^. Agricultural land is cultivated on individual farms, which structure is fragmented. The average farm in 2020 held 8.77 ha, while the national average was 11.04 ha^91^. According to statistical data, farms from 5 to 30 ha dominate covering 61% of the area of ​​all farms in the voivodeship (compared to 44.5% in Poland). In the period from Poland’s accession to the European Union, in the Mazovia Voivodeship, as in the rest of Poland, there was a relatively intense concentration of farms. However, small family farms still constitute the basis of agricultural production and the rural landscape.

The voivodeship mainly covers the historical–geographical region of Mazovia. It includes small areas of historical Lesser Poland, Podlasie, Kujawy and Dobrzyń Land. The fact that most of the Mazovia Voivodeship belongs to the Central European Lowland determines its lowland character. Small fragments of the eastern part of the voivodeship lie within the East Baltic-Belarusian Lowlands, whereas the southern part is within the Polish Uplands. River valleys such as the Vistula, Narew, Bug and Pilica are prominent in the landscape. In terms of climate, conditions favorable for recreation prevail from March to the end of October^68^.

At the end of 2019, the legally protected area of special natural values accounted for 29.7% of the voivodeship’s area. Compared to 2010, it increased by 2600 ha and the network of areas protected under the Natura 2000 program also expanded. Similarly, the forest cover ratio increased by 0.8 p.p. in 9 years and in 2019 forests occupied 23.5% of the voivodeship. Scenic landscape parks cover 173,297 ha (6.6% of the national scenic landscape park area) and the area of protected landscape areas covers 836,067 ha (11.9% of the national protected landscape area)^89^.

In recent years in the Mazovia Voivodeship, there has been a decrease in the level of emission of the main dust and gas pollutants (without carbon dioxide) generated in factories, which are particularly damaging for air quality. The degree of reduction of dust pollution reaches almost 100% and of gaseous pollution almost 80%. The main source of air pollution is the emission from the industrial sector (energy, production)^89^.

### Suitability of selected types of land use for various types of recreational behavior

To determine the rural and suburban areas with potential for various forms of recreation, a matrix of recreational behavior in rural areas was developed, taking into account the types of land use. In the matrix (see Supplementary Table S3), recreational behaviors are divided into two groups according to the dynamics of movement: static behavior (which can be practiced in a relatively small area) and dynamic behavior (related to movement in space). Each distinguished activity has been assigned the time of the year when it can be undertaken and the type of land use on which it can take place.

The matrix allows the determination of the features necessary for the area to be suitable for recreational behaviors, including those that were particularly popular during the pandemic (compare Supplementary Table S1). It played a role in defining the criteria and parameters to assess recreational potential in Mazovia Voivodeship.

### Recreational potential index

An original recreational potential index (RPI) was developed to assess the potential of rural and suburban areas to perform tourist and recreational functions. The index comprises four criteria based on a literature review, considering the conditions of reduced mobility and social distancing caused by the pandemic: landscape values and socio-economic conditions (K1); environmental protection (K2); air quality (K3); transportation accessibility (K4). The individual criteria have been characterized by universal indicators, for which the evaluation parameters were adjusted to the conditions of the Mazovia Voivodeship considering its characteristic features (see Table 1).

**Table 1:** Criteria for assessing tourist and recreational potential (Source: Own elaboration).

Criterion	Sub-criterion	Indicator	Parameter (established for Mazovia Voivodeship)	Data source
Landscape values and socio-economic conditions (K1)	Population density	W1—Population density (people/km^2^)	80 people/km^2^	Statistics Poland
	Farm size/area structure of agricultural holdings	W2—Share of farms greater than 15 ha in the total agricultural land in the commune [%]	> 40%	Statistics Poland, ARMA
	Agriculture land structure	W3—Share of grasslands (meadows and pastures) in the total agricultural land in the commune [%]	> 30%	Statistics Poland, LBR
	Forests area	W4—Share of forests in the commune area [%]	> 30%	Statistics Poland, LBR
	Surface water area	W5—Share of surface water in the commune area [%]	> 3%	Statistics Poland, LBR
	Income structure of population	W6—Indicator G—tax income in commune	< 1000	Statistics Poland
	Landscape diversity	W7— Shannon diversity index	> 0.54	LBR
Environmental protection (K2)	–	W8—Share of legal forms of nature protection in the commune area [%]	> 33%	GDEP
Air quality (K3)	–	W9—PM2.5 dust concentration in the summer season [μg/m^3^]	< 20 μg/m^3^	AIRLY
Transportation accessibility (K4)	–	W10—Commune’s accessibility from powiat cities with different means of transport	> 60 min (Warsaw)	Open Street MAP
			< 30 min (other powiat cities)	

#### Characteristics of criteria for assessing the recreational potential

The first criterion (K1) concerns landscape values, land use and selected socio-economic conditions. Its purpose is to reflect the natural and cultural values of the area, including natural elements and those shaped by human activity. The criterion was described by seven sub-criteria expressed by indicators (Table 1) including those used by Drzewiecki^92^ to identify rural recreational space. They were population density, farm size and agricultural land structure, area of forests and surface waters, income structure of the population living in the area analyzed and landscape diversity.

The first sub-criterion was related to population density (W1) based on Drzewiecki’s methodology^92^. Considering the conditions of limited mobility during the pandemic, it was assumed that areas inhabited by fewer than 80 people per sq. km would be more attractive for recreation. We considered the average population density in the communes of the Mazovia Voivodeship, amounting to 58 people per sq. km in rural communes and 91 people per sq. km in urban–rural communes^93^.

The second sub-criterion referred to the size of farms, expressed by the ratio of the area occupied by farms greater than 15 ha to the total agricultural land in the commune (W2). A mosaic landscape, characterized by a fine division of fields, a variety of crops, the maintenance of slight ground levelling and the associated mid-field afforestation and bushes, as opposed to large monocultures, is perceived as more attractive for recreation in terms of aesthetics and biodiversity. At the same time, it enables direct contact between tourists and farmers. It was assumed that the share of farms with an area of more than 15 ha, not exceeding 40% of the total area of farms, is the most favorable for recreational potential. Farms with an area of less than 15 ha, assuming traditional plant and animal production, have a low income and the obtained products are allocated mainly or exclusively to the self-supply of the associated households. Such farms are still a characteristic feature of Polish agriculture and the reasons why such subsistence production occurs are changing. In most cases, in the past, this was an important supplement to the low income of the rural population (especially households of retirees), but currently, it is more for ecological or health reasons, or as a hobby. The outbreak of the COVID-19 pandemic prompted owners to keep and urban populations to purchase habitats with little agricultural land. Presumably, this phenomenon will continue in the coming years^89^.

The third sub-criteria included the structure of agricultural land expressed in terms of the share of grasslands (meadows and pastures) in the total ​​agricultural land in the commune (W3), land covered with forests (W4) and water (W5) in ​​the commune. Permanent grassland increases the recreational potential of the area as, unlike arable land, it remains more accessible to tourists. Moreover, since it is covered with long-flowering vegetation, which positively affects the senses, it is characterized by high landscape values. It also provides the opportunity to observe species of fauna and flora. Supplementary Table S3 shows the types of recreational activities that can take place on meadows and permanent pastures. This methodology assumes that at least 30% of meadows and pastures as a share of total area of agricultural land (W3) has a positive effect on tourism.

One of the more important trends observed during the pandemic is the ‘return to nature’, which manifested primarily in increased use of forests for recreational purposes^24,29^. The emergence of this trend is the result of many factors, but the most crucial role is played by social, psychological and mental considerations: an increase in ecological awareness and care for health, and a negative assessment of the living environment and ‘fatigue with civilization’^94^. In this context, the forest provides ideal conditions to meet individual needs and offers several pro-health values^34,95–97^. Additional factors favoring the intensive use of forests for recreational purposes are increasing amounts of free time and improved transport accessibility (development of individual motorization, public transport, construction of walking and biking routes)^94^. Supplementary Table S3 shows the types of recreational activities possible in forested areas. It was assumed that developing tourism and recreation functions requires a share of at least 30% forest land in the total area of the studied entity (W4).

Water reservoirs greatly diversify the landscape and create an opportunity to observe both common and unique species of animals. In rural and urban–rural communes in the Mazovia Voivodeship, land covered with water accounts for only 1.1% of the total area. Therefore, we assumed that a share of over 3% of surface water in a commune’s area is beneficial for recreation development (W5). Supplementary Table S3 shows the types of recreational activities that require surface water.

Another important sub-criterion influencing the attractiveness for tourism and recreation is the structure of the population’s income. In the Mazovia Voivodeship, the percentage of households obtaining over 50% of income from agriculture increases with the farm size, reaching two-thirds in the 15–20 ha group and almost 90% in the group of 100 ha and more. Additionally, the average area of agricultural land in an individual farm, where the primary household income comes from agriculture, is twice the national average and is characterized by much higher growth dynamics^89^. To synthetically determine the structure and amount of income in the commune, indicator G concerning the tax income in the commune (W6) was used. This indicator, in accordance with the Act on the revenues of local government units^98^, is calculated by dividing the amount of the commune’s tax income (real estate; agricultural; forest; means of transport; civil law transactions; personal income tax, paid in the form of a tax card; revenues from stamp duty; revenues from exploitation fees; participation in revenues from personal income tax and participation in revenues from corporate income tax) for the year preceding the base year by the number of inhabitants of the commune. In the Mazovia Voivodeship, the indicator of basic tax revenues per commune inhabitant adopted for 2021 was, on average, PLN 1,542 in rural communes and PLN 2,083 in urban–rural communes^99^. Furthermore, it is worth noting that the receipts from agricultural tax constitute only a small share of municipalities’ incomes: about 4–5% of their self-income and about 2% of total income^100^. Considering the above, it was assumed that communes with indicator G values below 1000 would have high recreational potential, as they encompass a small share of people who make a living from non-agricultural activities.

The last sub-criterion was the level of landscape diversity expressed by the Shannon Diversity Index (W7). The indicator was calculated according to Eq. (1), considering the following types of land: (1) agricultural land; (2) orchards; (3) meadows and pastures; (4) ponds and ditches; (5) forests and small wooded land; (6) flowing and standing waters; (7) recreation areas; (8) other lands including built-up land.

1 $$H= - \sum_{i=1}^{j}{p}_{i}log {p}_{i},$$

where ${p}_{i}$ is the percentage of the i-th type of land use in the commune’s area. The factors of landscape contrast and diversity are often used in research on the visual attractiveness of the area^54,101^. The Shannon index reaches the highest values when the share of land-use forms in a spatial unit is even, i.e. when all forms occupy the same area. On this basis, it is possible to distinguish communes where the landscape is likely to be a mosaic of arable lands, meaning it is not monotonous and more stimulating for tourists. In the Mazovia Voivodeship, the mean value of the Shannon index is 0.52. Therefore, it can be assumed that spatial units with higher values ​​are more attractive for recreation.

In order to establish the total measure of landscape values and socio-economic conditions, the W1-W7 indices were combined by summing up their values.

The second criterion (K2), referring to environmental protection, was expressed by the share of the area covered by legal forms of nature protection in the commune area (W8). The following were considered: (1) protected landscape areas; (2) scenic landscape parks; (3) natural-landscape complexes; (4) Nature 2000 sites. These forms fully allow tourist access and have less restrictive rules for recreational and development use compared to national parks and nature reserves^102^. Moreover, the aim was to identify less popular destinations for recreation purposes, hence the decision to omit the well-established national parks^5,14,56^. In the Mazovia Voivodeship, the median coverage of communes with forms of nature protection is 33%; it can be assumed that units with a share higher than the median value are more attractive for recreation.

The third criterion (K3) refers to atmospheric air quality. The cleanliness of the natural environment plays a decisive role in determining the possibilities of recreation in a given region^48,67,103^. Indicators of PM2.5 dust concentration was selected because of its huge impact on population health and the direct correlation with the incidence of COVID-19. Other air quality indicators such as sulfur dioxide (SO_2_), nitrogen dioxide (NO_2_), benzene (C_6_H_6_), ozone (O_3_), heavy metals and benzo(a)pyrene (B(a)P) in PM10 dust were omitted from further analyses. GIS technology was used to approximate the current state of PM2.5 dust content in the air in a given area. Measurement data provided by the Airly organization (www.airly.org) was downloaded for 318 air quality recording stations in the Mazovia Voivodeship. Measurements in Airly databases are presented in real-time or in a historical form (e.g. from the last 24 h). To validate the results, the daily and monthly average dust content was calculated for each measuring station. In turn, the spatial location of their stations was obtained by vectorization based on Web Map Services (WMS). Then, based on the station’s quantitative data and spatial location, potential surfaces were generated, presenting the approximate state of atmospheric air quality in the studied area. These were obtained by conducting the interpolation process using deterministic and stochastic methods. Among many variants, the Inverse Distance Weighted (IDW) method was selected, which gave the most realistic results. Based on the obtained map of PM2.5 dust concentration in the summer season (W9) in the Mazovia Voivodeship, the average value of pollution was calculated for each commune.

The fourth criterion (K4) concerns transportation accessibility. When choosing a place for relaxation in a pandemic, accessibility by various means of transport is of great importance. The need to limit contact with other people meant that some residents chose individual car transport^9–12^. However, public transport, such as high-speed city rail, still plays an essential role in suburban transport. In the era of a pandemic and limited in mobility, residents increasingly began to choose alternative means of transport that favored physical activity, such as a bicycle^10^. Therefore, to determine the accessibility index of communes from powiat cities with different means of transport (W10), two variants of travel and time constraints were adopted:

- individual car transport within 30 min from the central point of powiat cities and 60 min from the central points of Warsaw districts
- transport by suburban railway within 30 min from the station within powiat cities, 60 min from Warsaw Central Railway Station and 60 min by bike from the destination station.

In the case of Warsaw, double travel time was assumed, both by individual and public transport, because of the large number of inhabitants and the size of the city.

In the study of the accessibility of individual transport, the road network was used, identified via OpenStreetMap and the Database of Topographic Objects. The data were supplemented with the permissible maximum speed considering road class, number of lanes and speed limits resulting from the location in the built-up area. The layers of railroads and railway stops from OpenStreetMap were used to assess the transport accessibility of the suburban railway. Again, the data were supplemented with the travel time from individual stations located within powiat cities. In this case, the accessibility of the areas was extended by bicycle transport, assuming a journey of 60 min with an average speed of 15 km/h. The prepared data were used to perform a network analysis in GIS software and the ranges of the transportation accessibility of suburban areas by various means of transport were obtained. The final indicator of accessibility for each commune was determined as a number representing the sum of powiat cities and means of transport, which can be used to get to the area of a given commune, divided by the highest value of accessibility.

#### Calculation of recreational potential index

Before determining the RPI, the individual criteria were normalized to ensure comparability of results. Equation (2) was used to obtain a uniform range of index values (from 0 to 1). In the case of the criterion of air quality (K3), it was necessary to apply Eq. (3) because PM 2.5 dust pollution is detrimental to recreational potential (the higher the concentration, the lower the attractiveness of the area).

2 $${z}_{i}=\frac{{x}_{i} - min \{{x}_{i}\}}{max \{{x}_{i}\}- min \{{x}_{i}\}}, i=1,\dots ,n$$

3 $${z}_{i}=1-\frac{{x}_{i-} -\mathrm{min}\left\{{x}_{i}\right\}}{\mathrm{max}\left\{{x}_{i}\right\}-\mathrm{min}\left\{{x}_{i}\right\}}, i= 1,..., n$$

RPI is a weighted arithmetic mean calculated according to Eq. (4).

4 $$RPI=\frac{{\sum_{i=1}^{4}{K}_{i}\times w}_{i}}{\sum_{i=1}^{4}{w}_{i}}, i \in [\mathrm{1,4}]$$

where ${K}_{i}$ is the value of i-th criterion and ${w}_{i}$ is the weight of i-th criterion.

Criterion weights were determined by the pairwise comparison method (Analytic Hierarchy Process (AHP)). In the first step, all authors individually assessed the significance of the criteria, comparing them in pairs according to the following principle: the more important criterion is assigned a value from 2 to 9 and the less important criterion is assigned a value from 1/9 to 1/2. In the case of equal significance, the criteria receive a grade of 1. On the basis of six pairwise comparisons, six sets of weights were determined, from which the arithmetic mean for each criterion was then calculated^104^, which was the final weight of the criterion (see Table 2).

**Table 2:** Criterion weights for RPI calculation (Source: own elaboration).

Criterion ${{\varvec{K}}}_{{\varvec{i}}}$		Weight ${{\varvec{w}}}_{{\varvec{i}}}$
K1	Landscape values and socio-economic conditions	0.32
K2	Environmental protection	0.23
K3	Air quality	0.26
K4	Transportation accessibility	0.19

## Assessment of the tourist and recreational potential of rural and suburban areas of the Mazovia Voivodeship for individual short-term tourism in pandemic conditions.

The developed methodology for designating areas allocated for tourist and recreational functions (METPRET) located in rural and suburban areas was tested in the Mazovia Voivodeship.

### Assessment results according to the criterion of landscape values and socio-economic conditions (K1)

One of the methodological assumptions is that the adopted indicators are constant and can be implemented in areas with different conditions; however, the selection of parameters depends on the specificity of the area to be assessed. Therefore, the parameters discussed below consider only the characteristics and specificity of the Mazovia region.

The research results based on the analysis of the K1 criterion are presented below while maps of its sub-criteria are shown in Supplementary Figures S2–S8. The summary of scoring of K1 criterion is presented in Supplementary Table S4 as well as on a map in Supplementary Figure S9.

#### Sub-criterion W1: population density

The Mazovia Voivodeship is characterized by a diversified population density (see Supplementary Fig. S2). The highest values of the indicator (80 persons per sq. km and above) were noticed in the ring of communes located near the capital city. The second-largest cluster of communes with high values of the indicator is located near the city of Radom. Moreover, high values are characteristic for single communes located around major cities like Płock, Żuromin, Ostrołęka, Pułtusk, Wyszków, Łochowa, Brok, Siedlce, Pilawa, Garwolin, Żelechów, Kozienice, Zwoleń, Lipsk, Białobrzegi and Warka. The remaining 209 communes, which cover over 75% of the total area of the voivodeship, are inhabited by less than 80 persons per sq. km.

#### Sub-criterion W2: farm size

The area of agricultural land in the vast majority (88%) of communes is dominated by farms smaller than 15 ha (see Supplementary Fig. S3). All communes located in the southern part of the voivodeship (south of Warsaw) and most communes to the east and west of the capital have such a character. In the northern and north-western part of the voivodeship and on its eastern border, there are communes in which the structure of agricultural land includes more than 40% of farms with an area above 15 ha.

#### Sub-criterion W3: agricultural land structure

In the structure of agricultural land, a compact area of 10 communes in the north-west (mainly in the Mławski powiat) and 16 in the north-east in the vicinity of Ostrołęka and Przasnysz stand out (see Supplementary Fig. S4). These communes are characterized by a high (30% and above) share of permanent grassland in the commune’s agricultural area. High values of the indicator are also characteristic for communes located near Warsaw (near its north-eastern border) and further on in that direction. High indicator values were also noted in five communes (Kampinos, Leoncin, Czosnów, Wieliszew, Brochów), located west of Warsaw along the Vistula River corridor. A high proportion of permanent grassland is found in single communes east of the voivodeship. However, in most communes (79%), grassland share in the agricultural land structure does not exceed 30%. Among them are the so-called strategic feeding areas of the Mazovia region, which are the basis of agricultural production^105^.

#### Sub-criterion W4: forest area structure

Municipalities with a forest share value above 30% constitute 23% of all municipalities in the voivodeship (see Supplementary Fig. S5). Larger concentrations are found in the northern part (e.g. the area of Biała Forest) and to the west and south-east of Warsaw. These are communes located in the ecological corridor of the Vistula River together with the area of the Kampinos Forest and the Kozienice Forest. High values of the indicator are also characteristic of single communes located at the western and south-western borders of Mazovia (Gostynin-Włocławek Forests and a part of the Świętokrzyska Primeval Forest).

#### Sub-criterion W5: area covered with surface water

Communes that obtained the highest values for the indicator describing the share of water in their area (3% and more) constitute 19% of the communes in the Mazovia Voivodeship (see Supplementary Fig. S6). The highest values of the indicator are typical for communes situated in the direct vicinity of watercourses. Communes located along the Vistula River and in the vicinity of the Narew and Bug rivers stand out. The landscape of the river valleys is characterized by a band-line structure and a great variety of ecosystems. These are also areas of high natural value and thus may have high tourist and recreational potential.

#### Sub-criterion W6: income structure

A vast majority of communes in the voivodeship (217) achieved high values of the indicator G (above 1000). Communes characterized by lower values are located further away from Warsaw and other larger cities in the area under study. There are 97 communes with indicator G values below 1000 (see Supplementary Fig. S7).

#### Sub-criterion W7: landscape diversity

Landscape diversity was calculated using the Shannon Diversity Index, considering different land-use types. The north-western part of the voivodeship, as well as its eastern and south-eastern part, are characterized by low landscape diversity (see Supplementary Fig. S8). In contrast, communes along the Vistula River situated to the south and north of Warsaw and to the west of Radom achieved high values of the indicator. Furthermore, communes located in the northern part of the Mazovia Voivodeship scored above average. A diversified land use structure in these areas can be explained by the fact that different land-use forms occupied similar areas.

The diversified rural landscape comprises forest complexes, a mosaic of fields, mid-field afforestation and natural and semi-natural vegetation enclaves, thus having a good ecological structure. The areas with high Shannon index values have potential visual values arising from a diversified landscape. They are likely to be attractive for many recreational activities listed in the recreational behavior matrix in Supplementary Table S3.

### Assessment results according to the criterion of environmental protection (K2)

The share of areas covered by legal forms of nature protection in communes of the Mazovia Voivodeship is diversified (see Supplementary Fig. S10). There are 44 municipalities with the highest indicator values (the proportion of nature protection forms is above 76%). At the same time, 15 municipalities are covered almost entirely (> 99%) by various forms of nature protection. However, 93 communes have less than 17% of their area covered with nature protection forms. In 56 communes, there are no such forms. Several clusters of communes with a high share of protected areas can be distinguished, i.e. north and south of Warsaw, as well as in the eastern and north-western part of the voivodeship.

Although areas occupied by national parks were excluded when determining the indicator, it should be noted that there are large areas under Nature 2000 protection (i.e. Biała Forest, Kozienice Forest and Kampinos Forest), which partially overlap with national parks. This explains the case of the Kampinos National Park to the north-west of Warsaw, which was not included in the assessment as a national park but as a Natura 2000 site.

High values of the indicator are also characteristic for communes located along the Vistula River, which has been included in various forms of nature protection. These areas constitute an ecological corridor and have high tourist and recreational potential. The majority of communes are located here, which are entirely (over 99% of the area) covered by different forms of nature protection. In addition, areas along other river valleys (the Narew, Bug and Pilica valleys) protected under the Nature 2000 system stand out as well as communes located in the north-west covered with protected landscape areas.

### Assessment results according to the criterion of air quality (K3)

It can be observed that the admissible contents for PM2.5 dust is not exceeded in accordance with the Regulation of the Minister of Environment on levels of certain substances in ambient air^106^ (see Supplementary Fig. S11). PM2.5 is considered the most harmful to human health. For the summer months, when outdoor recreation is usually practiced, levels of 25 µg/m^3^ (the permissible average annual level) are generally not exceeded. It should be noted that these are the standards in force in Poland, but the standards set by the World Health Organization or other European countries are much stricter. According to WHO, long-term exposure to PM2.5 concentrations higher than 10 µg/m^3^ results in increased mortality due to respiratory and circulatory diseases. In the case of this criterion, it was considered that the recreation practiced in these areas will be short-term and it does not pose a direct threat to human life and health across the whole Mazovia Voivodeship. Despite the lack of exceedances of admissible contents of PM2.5, a point assessment for communes was applied and the scores were assigned to appropriate classes using the cartogram method.

### Assessment results according to the criterion of transportation accessibility (K4)

The Mazovia Voivodeship communes are very diverse in terms of transportation accessibility (see Supplementary Fig. S12). The highest score was only obtained by 8 out of 317 communes (2.5%). The Piastów commune saw the maximum number of powiat towns from which the commune (14); other communes with the highest criterion value are accessible from 11–13 powiat towns. All these communes are directly adjacent to Warsaw.

There are 12 communes (3.8%) with a score of 0.75 (moderate and good) and on average, they are accessible from eight to nine powiat towns. These are mainly communes bordering Warsaw to the west. Around 32.8% of the communes gained a score of 0.5 (poor and average), being accessible from four to seven powiat towns. These are located in the direct vicinity of Warsaw and the other largest powiat towns. Communes with the weakest transportation accessibility (score 0.25) constitute approximately 60.9% of all and are accessible only from two powiat towns.

Examining the spatial distribution of all analyzed communes, there is a close correlation with the level of transportation development. The best accessible communes are those located closest to the capital city, to the west along the motorway and railway line, and to the north and south along national roads nos. 7 and 8. There is also a noticeable correlation between the accessibility of communes and the size of powiat towns. The best accessibility found in the central part of the voivodeship correlates with a high density of powiat towns with large populations and good development of transport facilities.

### Recreation potential index of communes in the Mazovia Voivodeship

**Figure 2:**
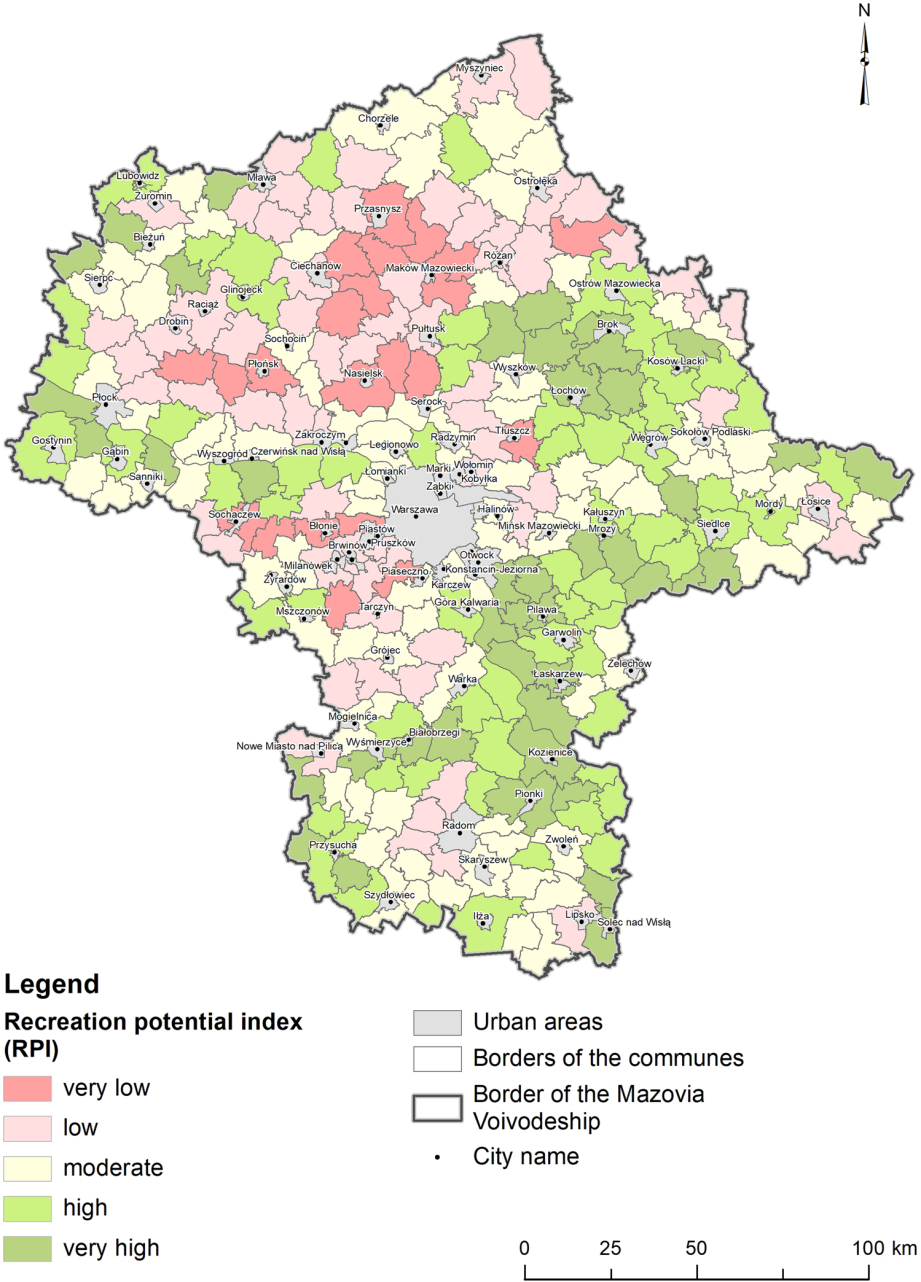
The tourism and recreational potential of communes in the Mazovia Voivodeship (Source: Own elaboration in ESRI ArcMap 10.8).

The evaluation ranges of the RPI are presented in Table 3, while the resultant map is in Fig. 2. The exact values of RPI obtained by individual communes in the Mazovia Voivodeship are presented in Supplementary Table S5.

**Table 3:** Score ranges of RPI.

RPI score	Recreation potential	Number of communes
0.146–0.261	Very low	22
0.262–0.357	Low	66
0.358–0.459	Moderate	86
0.460–0.580	High	62
0.581–0.834	Very high	43

The potential of communes in Mazovia to perform a tourism and recreation function is very diverse. Twenty-two communes obtained very low values of RPI, 66 obtained low values, 86 were in the range of medium values, 62 obtained high values and 43 communes obtained very high values.

The highest values of RPI occurred in communes located in the south, south-east and east of the voivodeship, as well as on its north-western border. Those communes are usually characterized by high coverage of various nature protection sites and thus achieved high values for the K2 criterion (environmental protection). Furthermore, it was estimated that the air quality was better than in other parts of the voivodeship.

The high value of RPI, particularly obtained by communes located in the south-east, in the vicinity of Warsaw, seems to be important from the point of view of shaping the spatial policy of commune to develop the tourist and recreational function. These include the following communes: Celestynów, Karczew, Sobienie-Jeziory, Pilawa.

Communes in the northern part of the voivodeship and south-west of Warsaw received the lowest RPI values because they scored low in the K1, K2 and K3 criteria. According to K1, their landscape was less rich in natural forms, i.e. forests, meadows and surface waters, and thus generally less diversified. The low share of natural elements resulted in smaller areas covered by various forms of nature protection (K2). These communes also had relatively higher levels of air pollution (K3). Regarding transportation accessibility, communes in the north scored lower than those in the vicinity of Warsaw.

Particularly attractive for recreation are communes located along major rivers, especially those in the vicinity of the Vistula River in the southern part of the voivodeship and the Bug River in the east. Due to their location in the Middle Vistula Valley, these communes are characterized by high biodiversity. For the most part, the river is relatively little transformed by man and thus has retained its natural rift character. Its sandy banks are regularly washed away during floods, allowing the preservation of unique fragments of willow and poplar riparian forests that are rare in analogous valleys of large European rivers. In the part of the valley outside the flood banks, valuable wetlands, oxbow lakes and remnants of old forests are scattered. These natural habitats are vital refuges for many European rare wetland birds and plants. Similarly, the Bug River valley contains many diverse natural elements, riverside deciduous forests, semi-natural flowering meadows, oxbow lakes, river dunes and swamps. In many places there is a considerable accumulation of rare species of plant and animal life characteristic for natural river valleys, which makes the Bug Valley a nature reserve of European importance^107^.

## Discussion

The present study aimed to develop a methodology (METPRET) for determining rural and suburban areas in the vicinity of cities that may fulfil tourist and recreational functions. The results show that it is possible to determine what characteristics such areas should have under pandemic conditions.

A review of the literature confirmed the initial research assumptions. In view of the increasing lack of areas for practicing various forms of physical activity in highly urbanized areas, more often, the functions of recreational zones of cities are being taken over by suburban areas to which both weekly and daily traffic is directed. It is predicted that new areas will be subject to such pressures^12,18,20,94,108^. In addition, the COVID-19 pandemic has demonstrated the need to revisit planning policies for designating new green spaces, including those in rural areas, to provide recreation and leisure areas for future generations^109^.

In the valorization of space for tourism and recreation, attempts are made to identify the features of the environment that are optimal for various forms of recreational activity^49^. The potential for tourism development is influenced by natural amenities, which primarily comprise landscape, climate, topography, and land use^74^. Although much research has been conducted on land suitability for tourism and recreation, including using the Rural Amenity Index^77^, it is still an open question of what and how many criteria and indicators to adopt so that results obtained can be compared and interpreted unambiguously^59^.

Analysis of recreational behavior during the pandemic^7,26,30,31,35^ has shown that there has been an increased interest in outdoor activities (especially those requiring little effort and specialistic equipment). This may largely be due to the fact, that for most people at that time, maintaining safety and social distance was the most crucial aspect^31,37,110^. Following the above research findings, a matrix of recreational behavior in rural and suburban areas was developed in relation to land use types (Supplementary Table S3). This matrix will help identify the characteristics of areas where, in the future, various tourism and recreational activities may be undertaken.

Use of the METPRET methodology to assess the potential of communes for individual short-term tourism makes it possible to select those communes that have both very high and high potential. It combines both criteria related to the values of the natural environment (which according to Potocka^49^ are most often considered in evaluations of tourism attractiveness) and agricultural land use to better reflect the specific character of the rural units under study. Such an approach makes it possible to assess the potential for tourism and to enhance the multifunctional development of rural areas while at the same time preserving the agricultural function in areas that are particularly allocated for this purpose. In addition, the selection of criteria was guided by the conditions caused by the COVID-19 pandemic, i.e. limited mobility of the population, social distancing, and the importance of atmospheric air quality. Dispersion of users outdoors, far from popular attractions, but close to home during short-term trips was assumed to be a primary objective.

The literature highlights that in valorization of a site for tourism and recreation, it is not uncommon for each landscape element to be treated as an independent value that is assessed separately^111^. Such an approach ignores the influence of interdependencies between individual landscape fragments on the actual tourism and recreational value. For this reason, the METPRET methodology proposes the use of the Shannon Diversity Index, which reflects the complexity of the landscape and the diversity of its forms. It is possible to select areas that are a mosaic of land uses occurring in similar proportions (lack of dominance of one land use type means lack of monotony). Such landscape is likely to be stimulating for recreation because the greatest natural richness is found at the meeting point of various landscape forms (water, forest, meadow, agricultural, built-up landscapes).

The main motive for choosing rural areas for recreation is the need for contact with a clean environment^112^, which has become particularly evident as a result of the COVID-19 pandemic^3,7,24–29^. When considering the suitability of land for recreation, information on the condition of the natural environment must be considered, as it affects comfort and health^58,113^. However, in previous assessments^48,53,58,67^ these factors were only marginally considered. Measurement data on air quality were often overlooked and, when they were included, assessments were limited by spatial scope. For example, air quality data provided by public statistics aggregated to the commune^53^ or voivodeship^67^ level were used, or other work relied solely on data provided by the poorly developed network of measurement stations of the Chief Inspectorate of Environmental Protection^48^. The proprietary METPRET methodology uses data with a higher degree of accuracy from a developed network of Airly measuring stations located in communes throughout the voivodeship, which provide real-time air quality information.

In previous evaluations for the purposes of tourism, transport accessibility was examined in a simplified way using indicators of the density of the road or railway network without analyzing the actual travel times for different means of transport. The METPRET methodology uses network analysis to calculate travel times to individual communes from powiat towns by different means of transport (car, rail, bicycle). Within its framework, a model of the transport network is created, considering the maximum travel speed on road segments concerning the road class and the number of lanes as well as restrictions resulting from the location in a built-up area and in relation to the suburban railway network (i.e. the travel time from particular stations located within the district towns).

During the development of the methodology, some limitations were encountered. The literature recommends that in shaping tourism demand in times of pandemic, in-country travel should be promoted, including trips to places with low tourist penetration and via individual and family use of individual transport^2^. However, studies on recreational behavior of Poles during the pandemic have focused primarily on destination choices for holiday travel^3,7,9,15,114,115^, without exploring urban residents’ preferences for short-term weekend and daily recreation. We argue that this issue should be further investigated, given the potential of rural areas to meet outdoor recreation needs and relieve pressure on existing green spaces in cities. In order to define specific requirements for the METPRET methodology, the mobility of Poles for recreational purposes should be analyzed.

Currently, no studies have been conducted in Poland on the time of travel to leisure and recreation areas. The few available studies are conducted for the needs of specific strategic documents drawn up at different levels of local government and are mostly limited to assessing the mobility of inhabitants of cities and suburban areas in the context of work and public transport development. The recreational behavior of Poles is assessed only in the context of domestic leisure and foreign travel^116^, omitting the aspect of distance and time spent on travel. Relying only on foreign studies may lead to unreliable results, as the manner and time of leisure travel are closely related to the size of urban centers, the intensity of suburban development, the remoteness and character of open areas, the development of means of communication and the road and rail network, as well as the wealth of residents and their leisure preferences^117,118^. Thus, it is necessary to base research on transport accessibility on the preferences of the inhabitants of a country or even a region, as well as on changes in population movements during a pandemic.

Another aspect that could be considered is the quality of travel. Research would need to explore whether the destination depends on the quality of the road infrastructure: the extent to which the destination that residents choose for recreational purposes is dictated by expressways, which provide fast and comfortable travel over long distances, or whether they choose local roads with less traffic regardless of their quality and capacity, or whether the direction of travel is unrelated to road infrastructure quality. Checking these issues would contribute to a more detailed criterion of transport accessibility and a more accurate reflection of the preferences of inhabitants of a given area.

It is also worth noting the uncertainty surrounding the relationship between air quality and morbidity during the pandemic. Currently available data do not allow a reliable estimate of the percentage of COVID-19 deaths that can be attributed to air pollution (PM10 or PM2.5). Calculations presented in publications^86,87^ should be approached with caution. COVID-19 survivors may be more susceptible to the harmful effects of breathing polluted air. The epidemiological studies mentioned earlier in the present paper indicated a correlation between air pollution and the course of disease and epidemics. However, it should be borne in mind that this analysis has many variables. Air pollution correlates with population density, so it can sometimes be difficult or even impossible to distinguish the influence of different factors. However, these relationships require further research and statistical analysis based on the increasing amount of available spatial data.

Finally, the authors see further possibilities for developing the METPRET methodology. One of the criteria that possibly should be introduced into the RPI is bioclimatic conditions, especially when the spatial scope of the analysis is to transcend a region and thus would involve a greater diversity in topography and the influx of air masses with different sources of origin. Climatic factors play a role in assessing recreational potential because certain weather conditions affect the human body implying disease symptoms, subject ailments, and complications in the health resort treatment^70^. They also shape leisure satisfaction. In Poland, according to the Comprehensive Weather Index for Recreation^68^, conditions for tourism are fairly uniform in the main tourist seasons (favorable conditions prevail in all regions from March to the end of October). Most of the country’s population lives in a low-stimulus type of climate occurring in the lowlands^72^. Travelling within this type of climate requires little or no adaptation by the human body. For these reasons, the RPI does not incorporate climate indicators. Nevertheless, the authors suggest preceding the assessment with an analysis of the bioclimatic zones for recreation in the country.

## Summary and conclusions

We proposed the METPRET methodology for determining rural and suburban areas in the vicinity of cities with the potential to perform tourism and recreation functions. It addresses the need to search for new areas for recreation and health restoration in limited mobility and social distancing conditions. The suburban and rural areas identified with METPRET methodology may serve as a proposal to expand the short-term tourism destination base while simultaneously contributing to relieving the pressure on urban leisure spaces.

The features that should characterize areas suitable for tourism and recreation in pandemic conditions were determined using the original Recreational Potential Index (RPI). The proposed set of four evaluation criteria (K1—landscape values and socio-economic conditions, K2—environmental protection, K3—air quality and K4—transportation accessibility) refers to the most critical assets from a pandemic point of view, when the main concern of users was to recreate outdoors, close to nature, in a clean environment, relatively close to home but far from other people. The advantage of the RPI is that it can be adapted to the specificity of the area to be assessed because evaluation parameters are set according to the conditions of the region. In the example presented, these are related to the environmental and socio-economic conditions of the Mazovia Voivodeship.

Areas characterized by high and very high RPI values are those covered by various forms of nature protection, with attractive landscapes (presence of forests, surface waters), no favorable conditions for the development of large-scale agriculture, clean air, and good transport accessibility (maximum travel time up to 60 min). The proposed methodology may be applied by local authorities when preparing planning documents for areas located in rural and suburban areas. In turn, a recreational activity matrix will help decide what kind of recreational base to build, including activities divided by the dynamics of movement and season concerning the type of land use.

## Supplementary Information

Supplementary Information.

## Data Availability

All data generated and analyzed during this study are included in this article and its Supplementary Materials.
