# Supplementary material for: Evaluating the potential of suburban and rural areas for tourism and recreation, including individual short-term tourism under pandemic conditions
Source: Sci Rep. 2022 Nov 27;12:20369. doi: 10.1038/s41598-022-24503-z (PMC9701685; doi:10.1038/s41598-022-24503-z)
Supplement: Supplementary file 1 — Supplementary Information. [file 41598_2022_24503_MOESM1_ESM.pdf]

Evaluating the potential of suburban and rural areas for tourism and recreation, including individual short-term tourism under pandemic conditions

Anna Bielska, Andrzej Szymon Borkowski, Adrianna Czarnecka, Maciej Delnicki, Jolanta Kwiatkowska-Malina, Monika Piotrkowska

Supplementary Material

Supplementary Figure S1. Schema of METPRET methodology (Source: Own elaboration in Microsoft Publisher Version 2209)

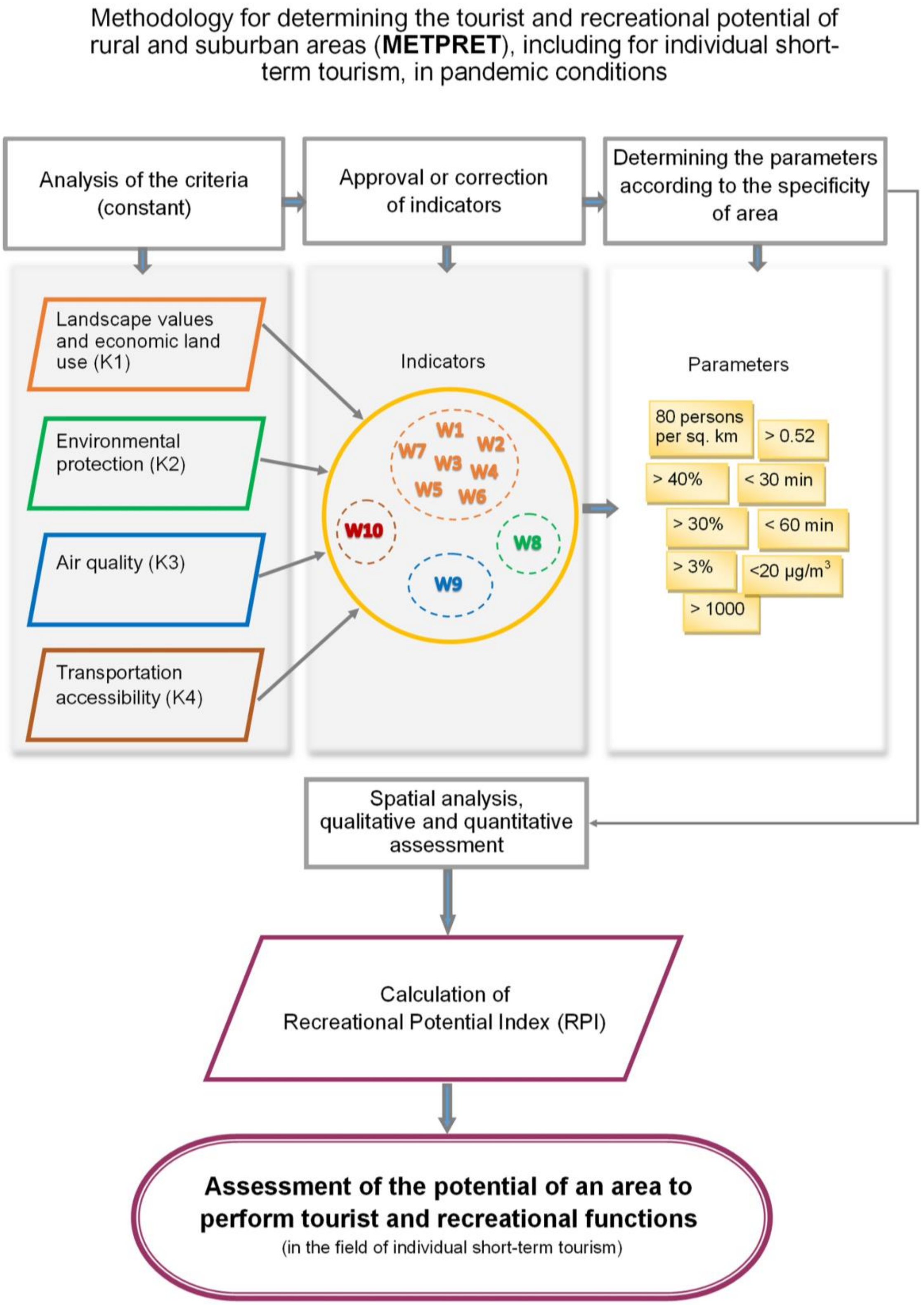

Supplementary Table S1. Changes in the types of outdoor activities during the COVID-19 pandemic (Source: Own elaboration based on multiple sources [1-4])

	Trend			Source
	Increase	No change	Decrease	
Type of activity	walking,<br>watching wildlife,<br>relaxing alone,<br>gardening, photography,<br>hiking,<br>jogging,<br>foraging	hunting,<br>boating,<br>rock climbing,<br>biking,<br>fishing	camping,<br>relaxing with others	1
	birdwatching or wildlife viewing, gardening, jogging	-	backpacking,<br>camping,<br>climbing,<br>downhill skiing or snowboarding	2
	jogging,<br>walking,<br>cycling	-	-	3
	gardening, hammocking,<br>jogging,<br>walking (outdoors),<br>birdwatching or wildlife viewing, playing with pets, collecting wild plants,<br>kite skiing/boarding, road cycling,<br>lawn games,<br>nature photography	-	mountaineering,<br>outdoor hockey,<br>participating in outdoor educational programmes,<br>camping,<br>rock climbing, swimming,<br>downhill skiing or snowboarding,<br>canoeing, backpacking	4

Supplementary Table S2. Criteria for tourist attractiveness assessment (Source: Own elaboration based on multiple sources [5–20])

Group of criteria	Criterion	Analysed feature	Indicator
Tourist values - sightseeing values	Landscape diversity	Scenic values, landscape contrast	Number of land cover types<br>Shannon Diversity Index
	Occurrence of natural elements	Uniqueness of landscape	Number of natural features (rocks and groups of rocks, valleys, river gorges, ravines, waterfalls, springs, caves, erratic boulders) in the spatial unit<br>Occurrence of unique landscape complexes
	Forms of nature protection	Ecological and cultural values, uniqueness of landscape	Percentage of area under forms of nature protection[1]<br>Number of nature protection forms<br>Percentage of area under reserves<br>Percentage of area covered by scenic landscape parks<br>Percentage of area covered by protected landscape area<br>Natural monuments per sq. km
	Occurrence of cultural elements (including folk culture)	Scenic values, cultural values, suitability for various forms of recreation	Density of architectural monuments, urban and rural complexes and landscape dominants in the spatial unit<br>Number of museums and open-air museums<br>Occurrence and prominence of places of national remembrance and battlefields<br>Occurrence of places of worship and pilgrimage<br>Presence of archaeological sites (legible in the landscape, adapted to tourism)<br>Presence of periodical cultural events
Tourist values - recreationaland specialized values	Land cover variety	Scenic values, suitability for various forms of recreation	Percentage of forests<br>Percentage of meadows and pastures<br>Percentage of arable land
	Relief diversity	Scenic values, suitability for various forms of recreation	Relative heights<br>Average slope<br>Relief factor
	Vegetation	Health-promoting qualities,<br>scenic values	Variety of the forest species composition<br>Types of plant communities
	Surface waters	Scenic values, suitability for various forms of recreation	Percentage of surface waters<br>Number of surface water types<br>Length of watercourses<br><br>For the purpose of assessing the suitability of water reservoirs for recreation:

[1] In Poland there are several legal forms of nature protection i.e., national parks, nature reserves, scenic landscape parks and protected landscape areas. Other small-scale forms of protection include ecological sites, documentation posts, natural-landscape complexes and natural monuments. Most of them allow recreation use.

			Lack of dense vegetation on the lake shoreline<br>Diversity of lake shoreline features (bays, beaches)<br>Percentage of area covered by the escarpment in the 250 m wide strip of lakeshore<br>Percentage of underwater slope in 100 m wide strip from the shoreline (zone where the slope exceeds 10°)<br>Depth of 100 m wide strip from the shoreline (percentage of area with a depth of up to 1 m)
	Mineral waters	Therapeutic qualities	Types and capacity of mineral springs
	Climatic conditions	Suitability for various forms of recreation	Length of the general recreation season (identified with the plant growing season)<br>Length of the bathing season (the period between the multi-year average of the occurrence and disappearance of water temperatures above 18°C)
	Bioclimatic conditions	Health-promoting qualities, suitability for various forms of recreation	Number of days with favourable temperature<br>Number of days with favourable weather types<br>Number of hours of sunshine<br>Number of days with snow cover<br>Presence of wetlands and marshes
State of the natural environment	Air quality	Health-promoting qualities	Exceedance of permissible air pollution levels<br>Pollution emission (thousand tons per sq. km)<br>Concentration of particulate matter PM10 and PM2.5
	Noise levels	Health-promoting qualities	Exceedance of permissible noise levels<br>Occurrence of significant noise emitters (e.g., transit lines)
	Water purity	Scenic values, suitability for various forms of recreation	First- and second-class waters<br>Discharge volume of industrial and communal wastewater
	Degree of urbanization	Health-promoting qualities, scenic values	Share of built-up land<br>Presence of industrial and storage zones
Site accessibility	Transport accessibility	Convenient access / ease of access	Length of public roads with hard surface in km per 100 km² of area<br>Length of the railroad lines in km per 100 km² of area
	Time availability	Flexible forms of access	No occurrence of restrictions in access to the facility or an area depending on the time of day and season of the year
	Information availability	Variety of ways and forms of	Signage in the form of information boards<br>Degree of popularization in guide literature and specialist publications

		providing information	Number of tourist guides, sightseeing tour operators
Tourist infrastructure	Tourist accommodation	Capacity and occupancy rate of accommodation base	Schneider's index (tourists in tourist accommodation per 1000 permanent inhabitants)<br>Defert's index (tourists in tourist accommodation per sq. km)<br>Charvat's index (number of overnight stays in relation to 100 inhabitants of the study area)<br>Density of bed places in the area<br>Number of beds per 1000 inhabitants<br>Number of accommodation facilities<br>Total capacity of accommodation facilities
	Tourist facilities	Number and variety of recreational facilities	Number of ski-lifts, cable cars, swimming pools, tennis courts, golf courses<br>Length of hiking trails, horseback riding trails, waterways, bike trails, ski trails, etc.

Supplementary Table S3. Matrix of recreational behaviour in rural areas in terms of land-use type (Source: Own elaboration)

No.	Dynamic of movement	Activity type	Season (S – spring, Sm – summer, A – autumn, W – winter)				Land-use type (M/P – meadow or pasture, F – forest, W – water, R – road)			
			S	Sm	A	W	M/P	F	W	R
1	Static	picnicking	●	●	●		●	●		
2		sunbathing	●	●			●		●	
3		hammocking	●	●	●			●		
4		birdwatching/watching insects	●	●			●	●	●	
5		nature photography	●	●	●	●	●	●	●	●
6		flying kites	●	●			●			
7		picking herbs and flowers	●	●			●	●		
8		catching butterflies/insects	●	●			●			
9		picking mushrooms		●	●			●		
10		burning a campfire	●	●	●		●			
11		camping	●	●	●		●			
12		flying drones	●	●			●			
13		flying remote-controlled aircrafts	●	●			●			
14		fishing		●	●	●			●	
15		hunting		●	●	●	●	●		
16		sky observation (clouds)	●	●			●		●	
17		sky observation (stars)	●	●		●	●			
18		winter/ice swimming				●			●	
19		reading	●	●			●	●	●	
20		listening to music	●	●	●		●	●	●	●
21		meditation	●	●			●	●	●	
22		yoga	●	●			●	●		
23	Dynamic	walking/walking a dog	●	●	●	●	●	●		●
24		Nordic walking	●	●	●	●	●	●		●
25		jogging	●	●	●	●	●	●		●
26		rollerblading	●	●	●					●
27		biking/cycling	●	●	●		●	●		●
28		skiing				●	●	●		●
29		quad riding	●	●	●	●	●	●		●
30		motorcycling	●	●						●
31		swimming		●					●	
32		horse riding	●	●	●	●	●	●		●
33		remote controlled boat driving		●					●	
34		kayaking		●					●	
35		driving pontoons		●					●	
36		boating / pedal boating		●					●	
37		stand-up paddle boarding		●					●	
38		yachting	●	●	●				●	
39		wakeboarding		●					●	
40		skating				●			●	

Supplementary Figure S2. Population density in communes of the Mazovia Voivodeship (Map of Sub-criterion no. 1 of landscape values and socio-economic conditions (K1)) (Source: Own elaboration in ESRI ArcMap 10.8 based on data from Statistics Poland and NRB)

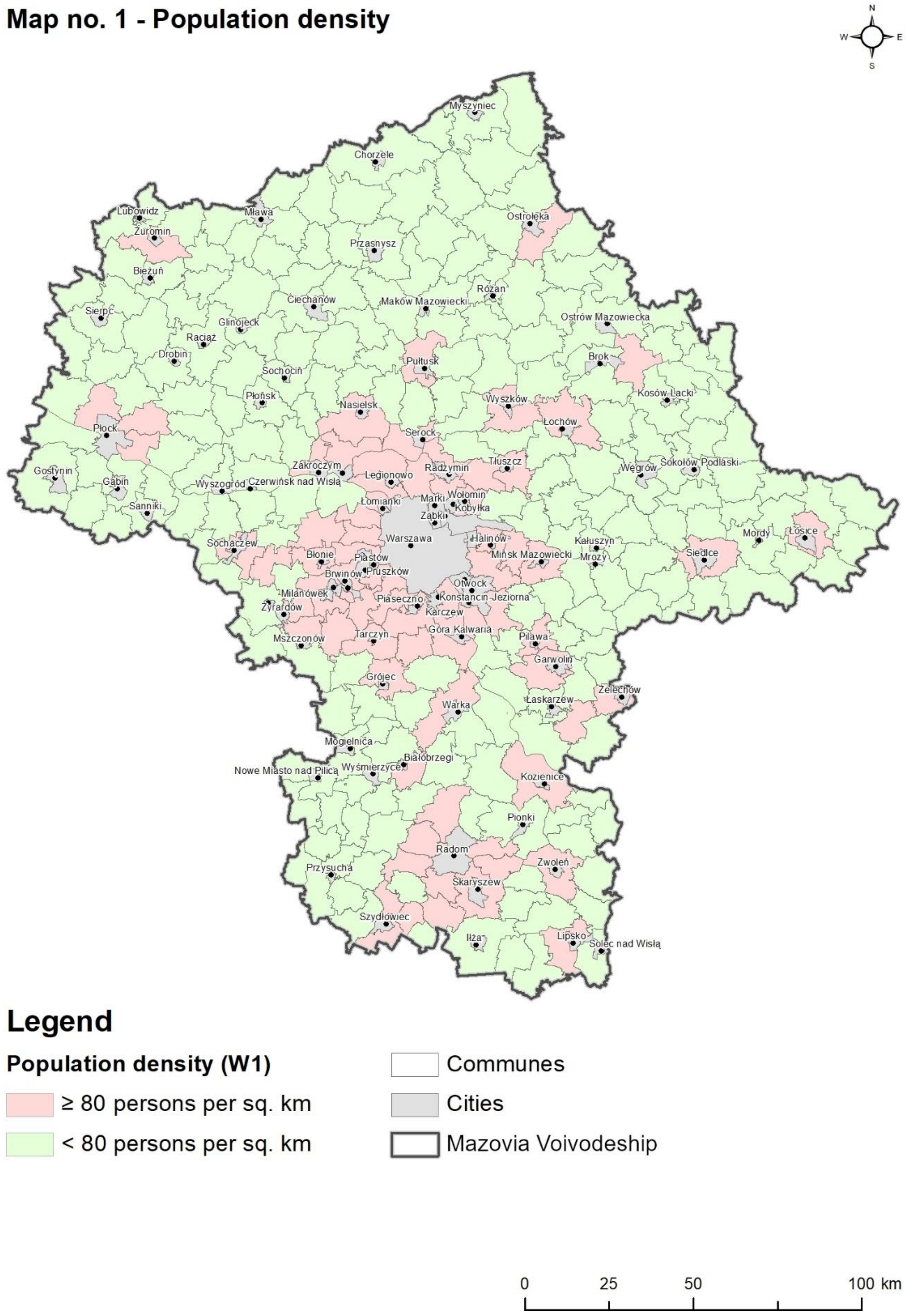

Supplementary Figure S3. Farm size in communes of the Mazovia Voivodeship (Map of Sub-criterion no. 2 of landscape values and socio-economic conditions (K1)) (Source: Own elaboration in ESRI ArcMap 10.8 based on data from Statistics Poland, ARMA and NRB)

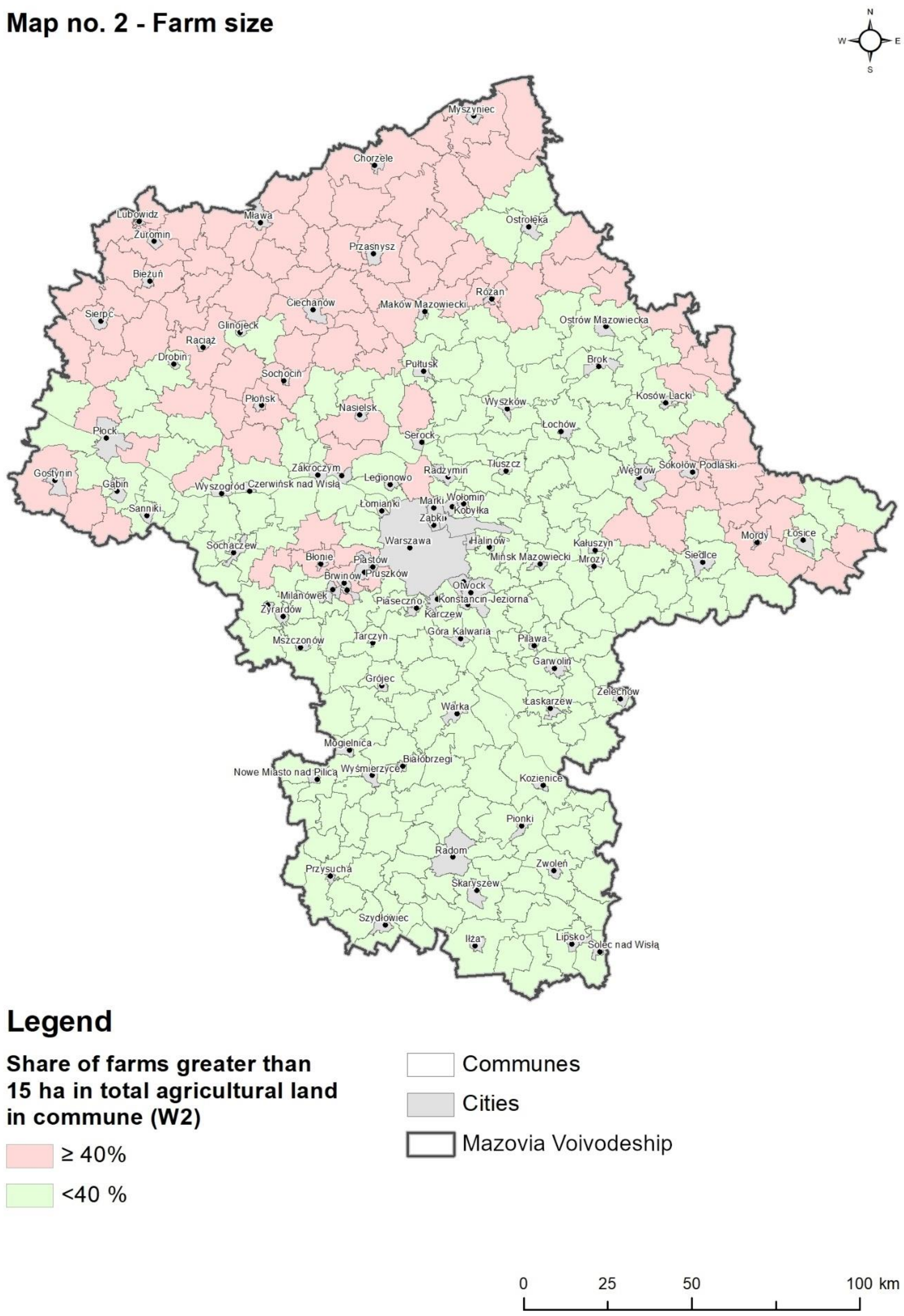

Supplementary Figure S4. Agricultural land structure in communes of the Mazovia Voivodeship (Map of Sub-criterion no. 3 of landscape values and socio-economic conditions (K1)) (Source: Own elaboration in ESRI ArcMap 10.8 based on data from Statistics Poland, LBR and NRB)

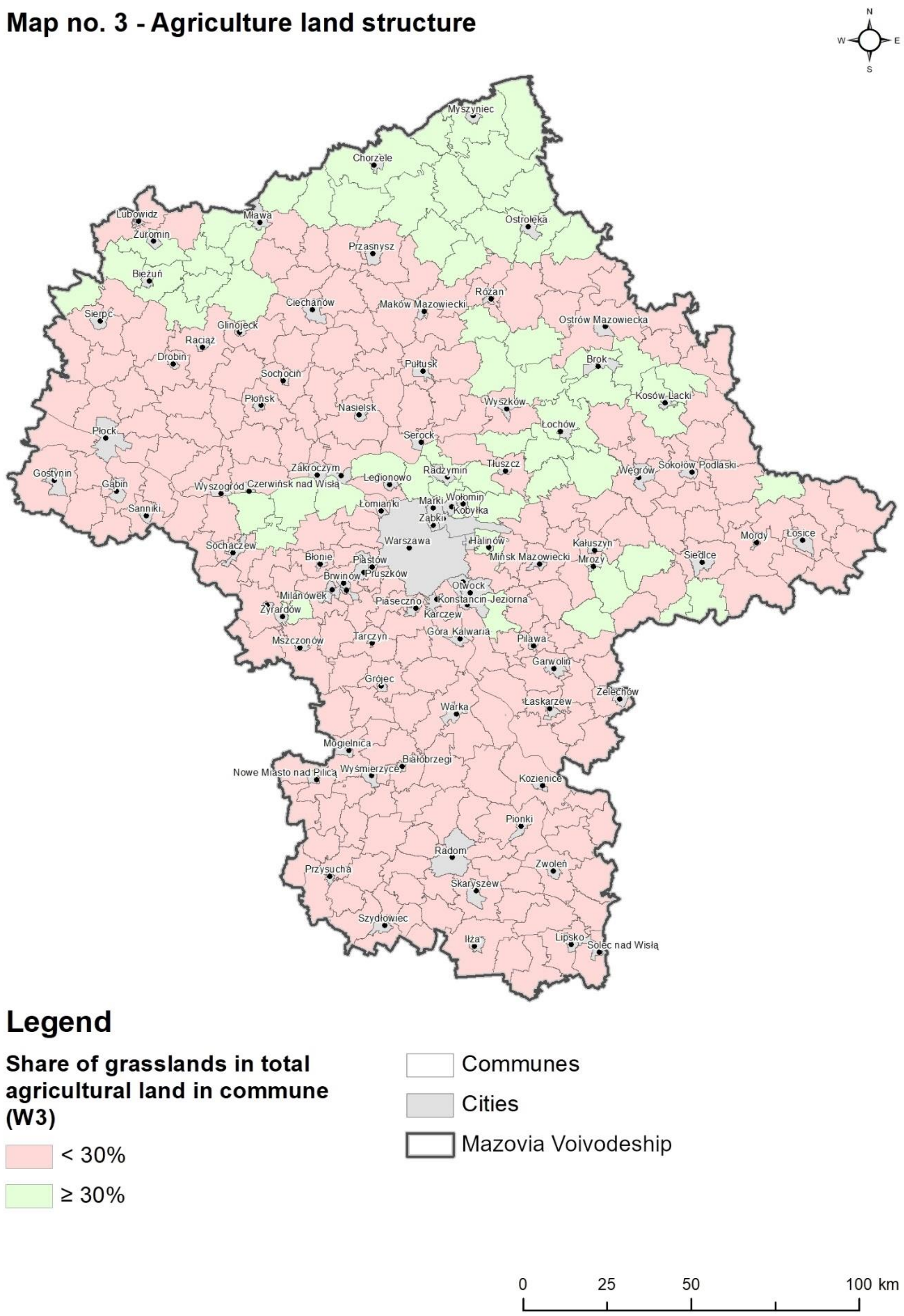

Supplementary Figure S5. Forest area in communes of the Mazovia Voivodeship (Map of Sub-criterion no. 4 of landscape values and socio-economic conditions (K1)) (Source: Own elaboration in ESRI ArcMap 10.8 based on data from Statistics Poland, LBR and NRB)

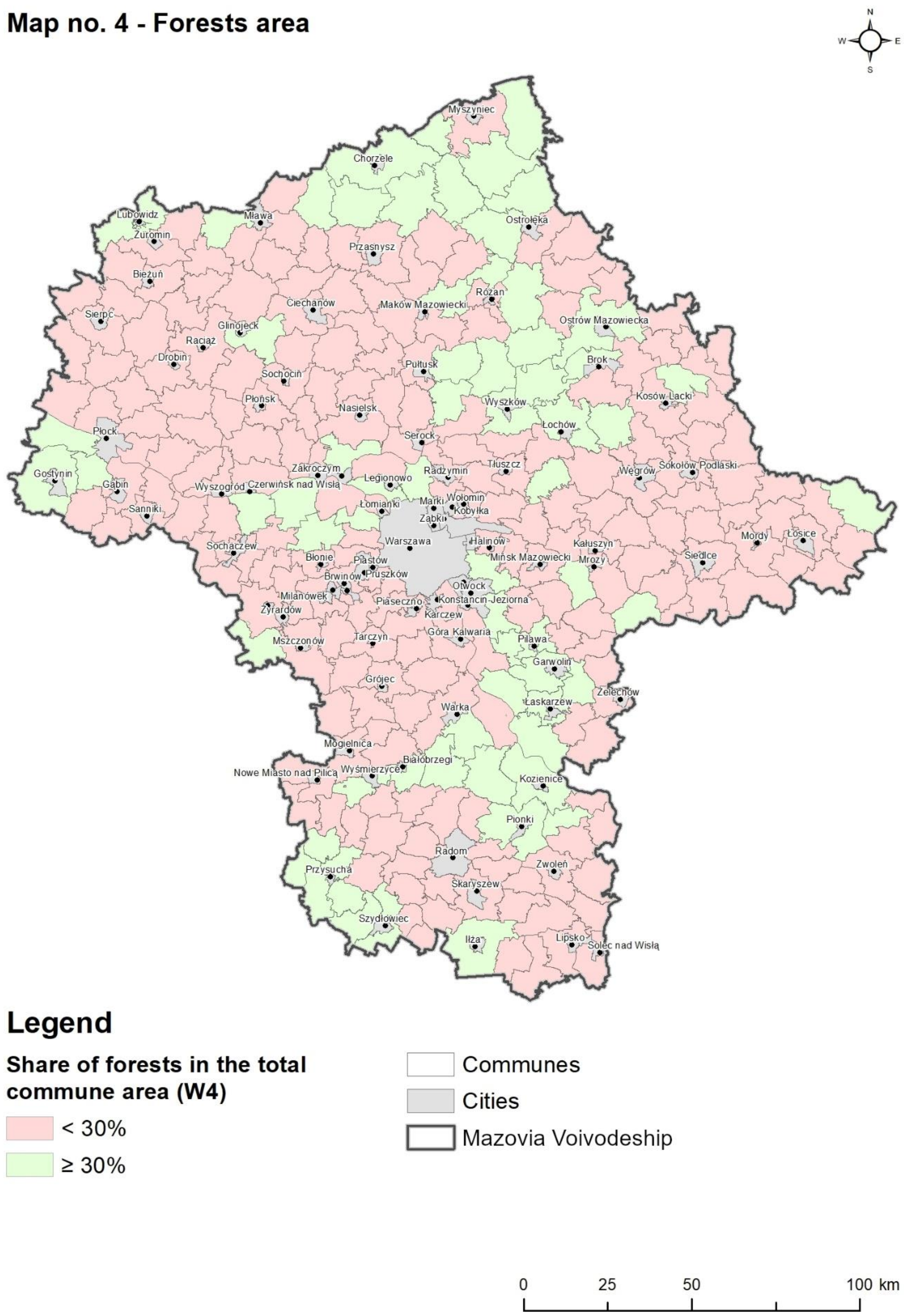

Supplementary Figure S6. Surface water area in communes of the Mazovia Voivodeship (Map of Sub-criterion no. 5 of landscape values and socio-economic conditions (K1)) (Source: Own elaboration in ESRI ArcMap 10.8 based on data from Statistics Poland, LBR and NRB)

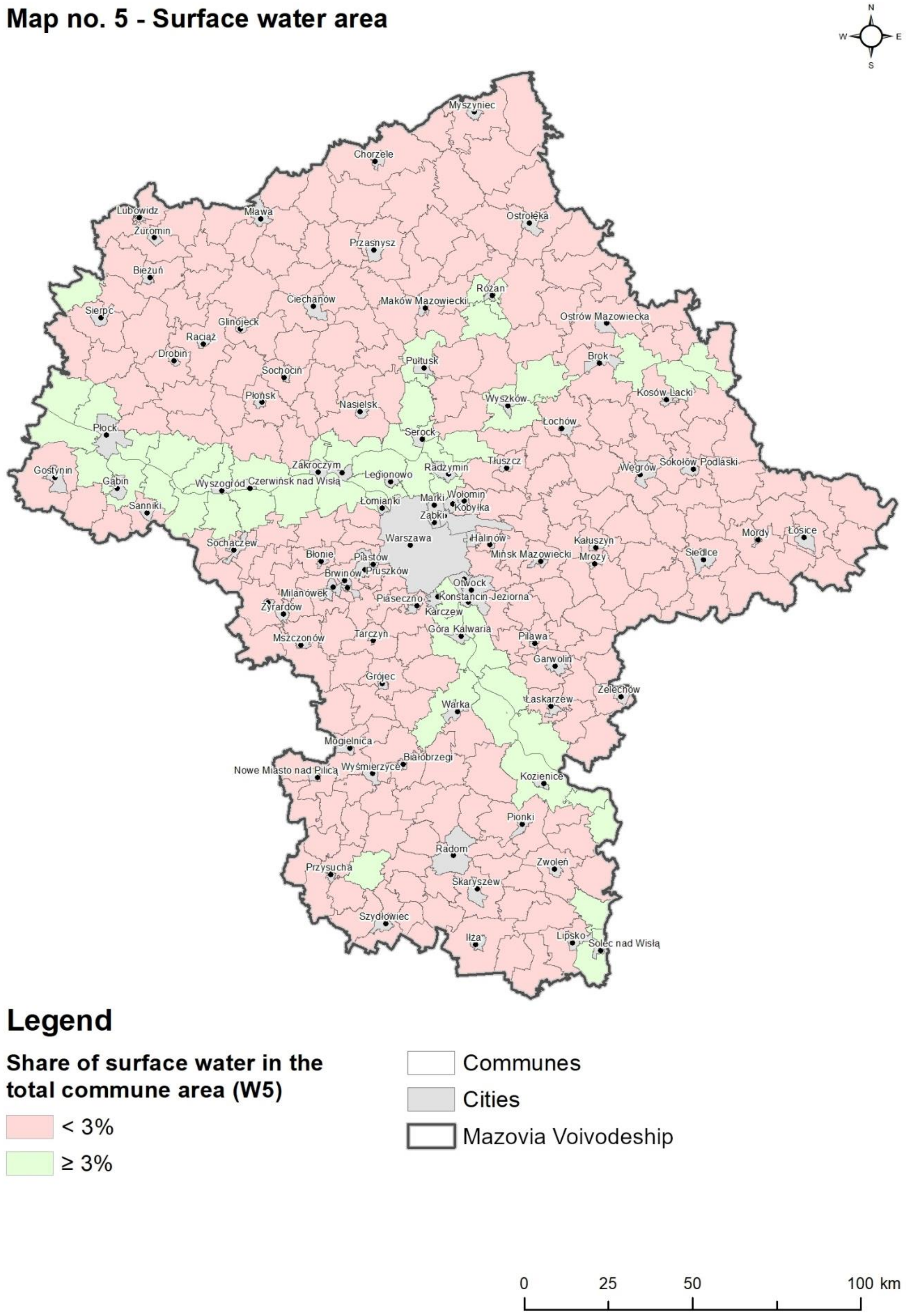

Supplementary Figure S7. Income structure of population in communes of the Mazovia Voivodeship (Map of Sub-criterion no. 6 of landscape values and socio-economic conditions (K1)) (Source: Own elaboration in ESRI ArcMap 10.8 based on data from Statistics Poland and NRB)

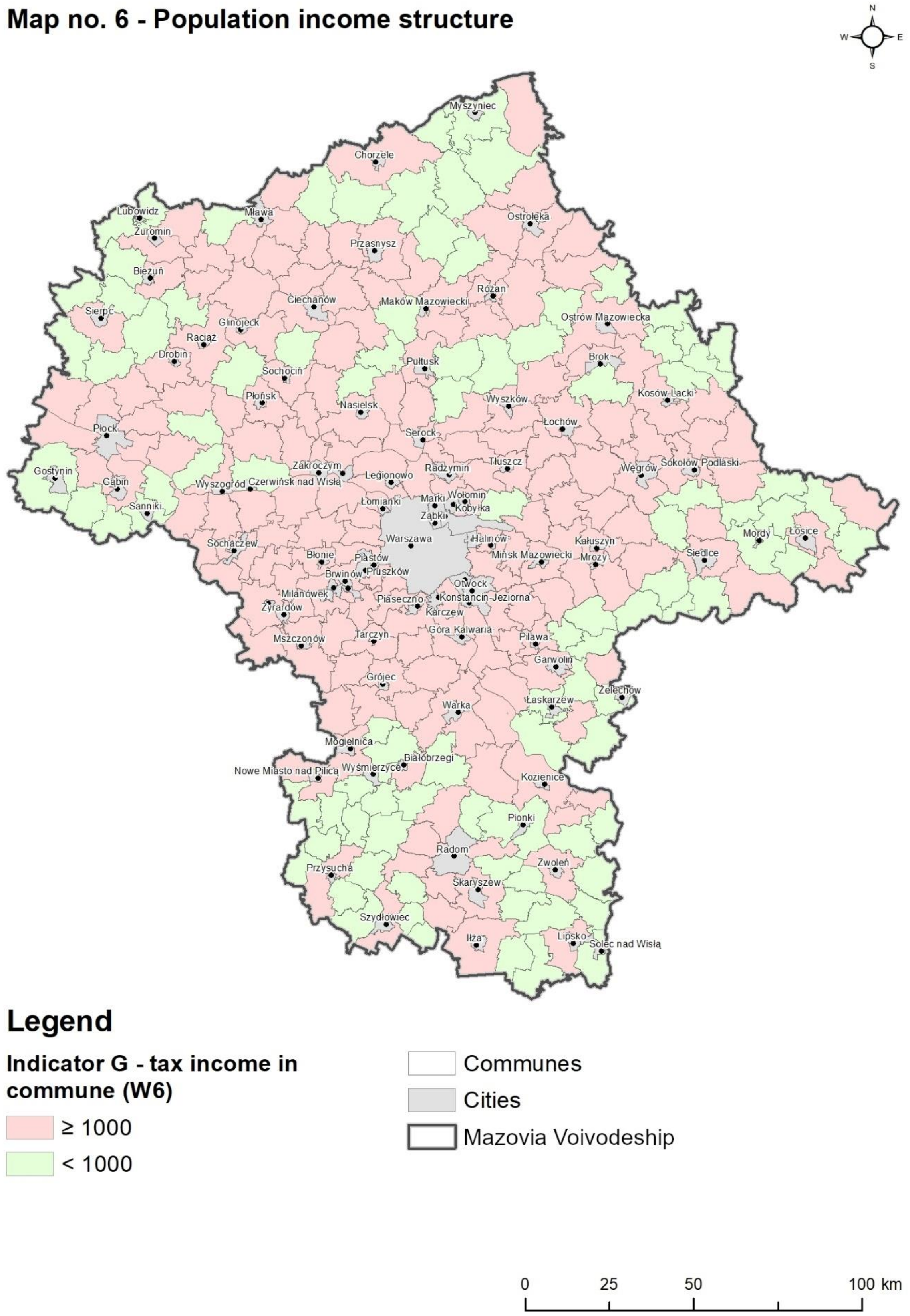

Supplementary Figure S8. Landscape diversity in communes of the Mazovia Voivodeship (Map of Sub-criterion no. 7 of landscape values and socio-economic conditions (K1)) (Source: Own elaboration in ESRI ArcMap 10.8 based on data from LBR and NRB)

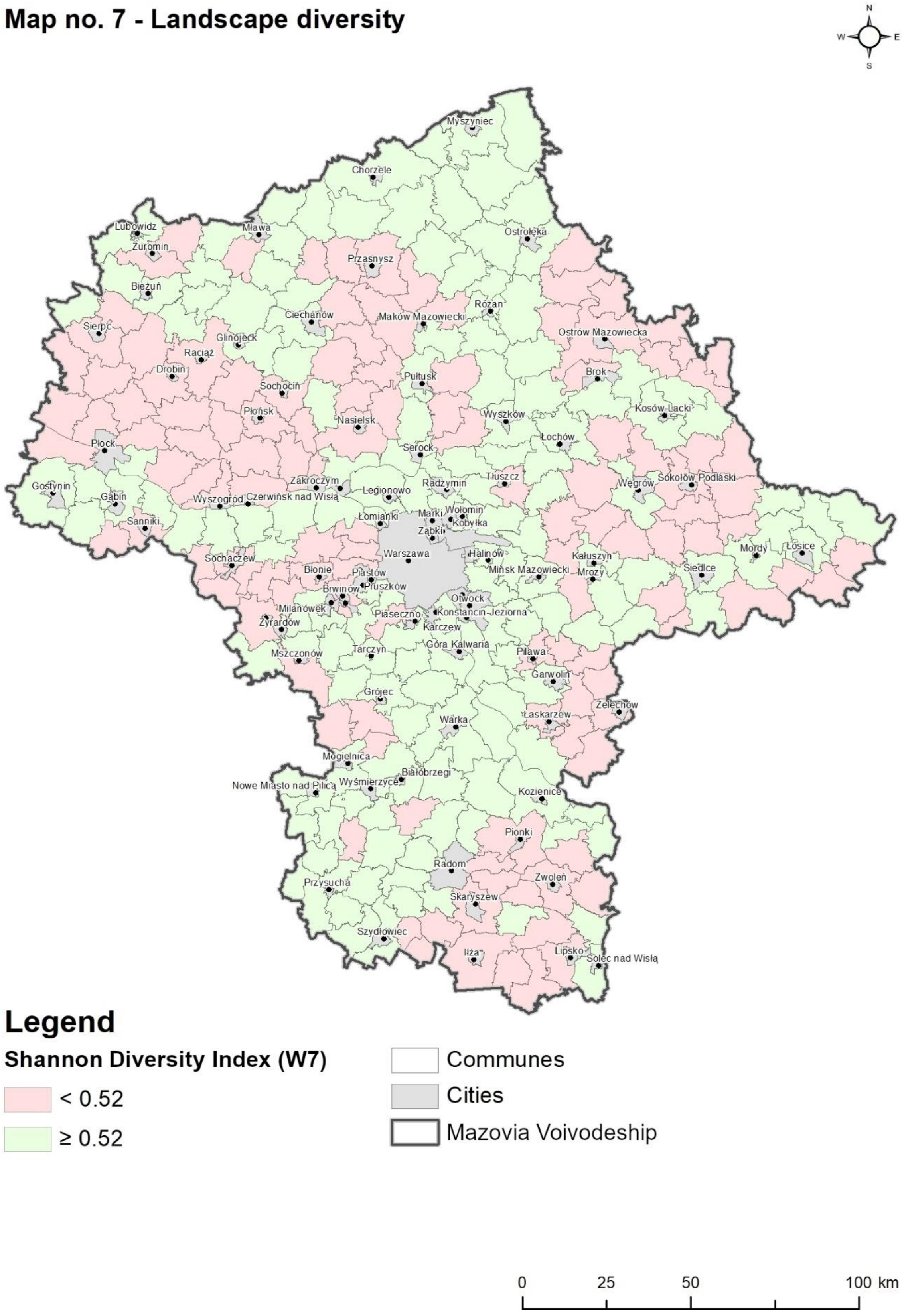

Supplementary Table S4. Scoring method of criterion Landscape values and socio-economic conditions (K1)

No.	Sub-criterion	Indicator	Parameter	Score
1	Population density	W1 – Population density (persons/km²)	≥ 80 persons per sq. km	0
2			< 80 persons per sq. km	1
3	Farm size	W2 – Share of farms greater than 15 ha in total agricultural land in commune [%]	≥ 40%	0
4			< 40%	1
5	Agriculture land structure	W3 – Share of grasslands (meadows and pastures) in total agricultural land in commune [%]	≥ 30%	1
6			< 30%	0
7	Forests area	W4 – Share of forests in the total commune area [%]	≥ 30%	1
8			< 30%	0
9	Surface water area	W5 – Share of surface water in the total commune area [%]	≥ 3%	1
10			< 3%	0
11	Income structure of population	W6 – indicator G - tax income in commune	< 1000	1
12			≥ 1000	0
13	Landscape diversity	W7 – Shannon Diversity Index	≥ 0.52	1
14			< 0.52	0

Supplementary Figure S9. Landscape values and socio-economic conditions (K1) in communes of the Mazovia Voivodeship (Source: Own elaboration in ESRI ArcMap 10.8 based on Sub-criteria no. 1-7 and NRB)

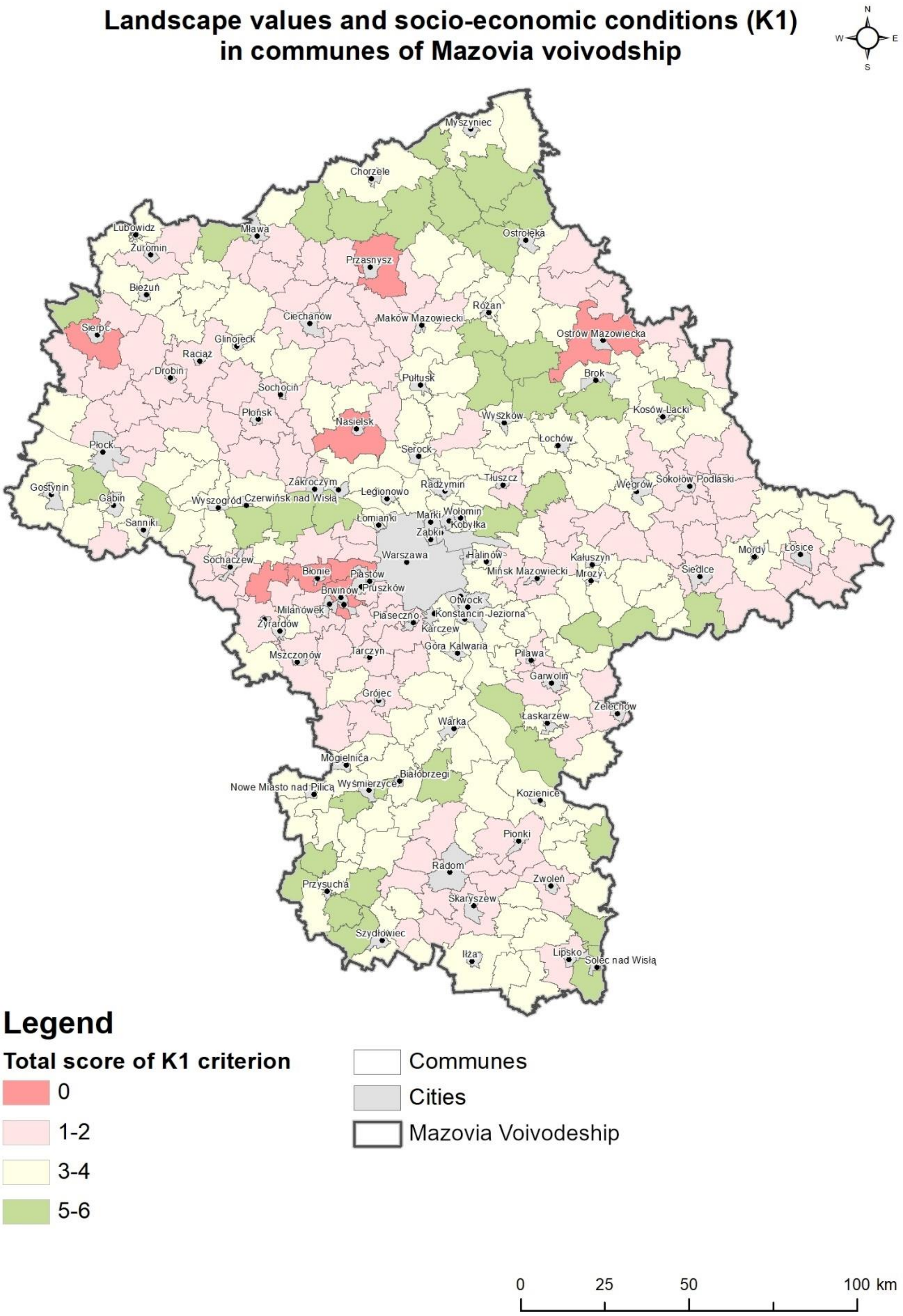

Supplementary Figure S10. Environmental protection (K2) in communes of the Mazovia Voivodeship (Source: Own elaboration in ESRI ArcMap 10.8 based on data from GDEP and NRB)

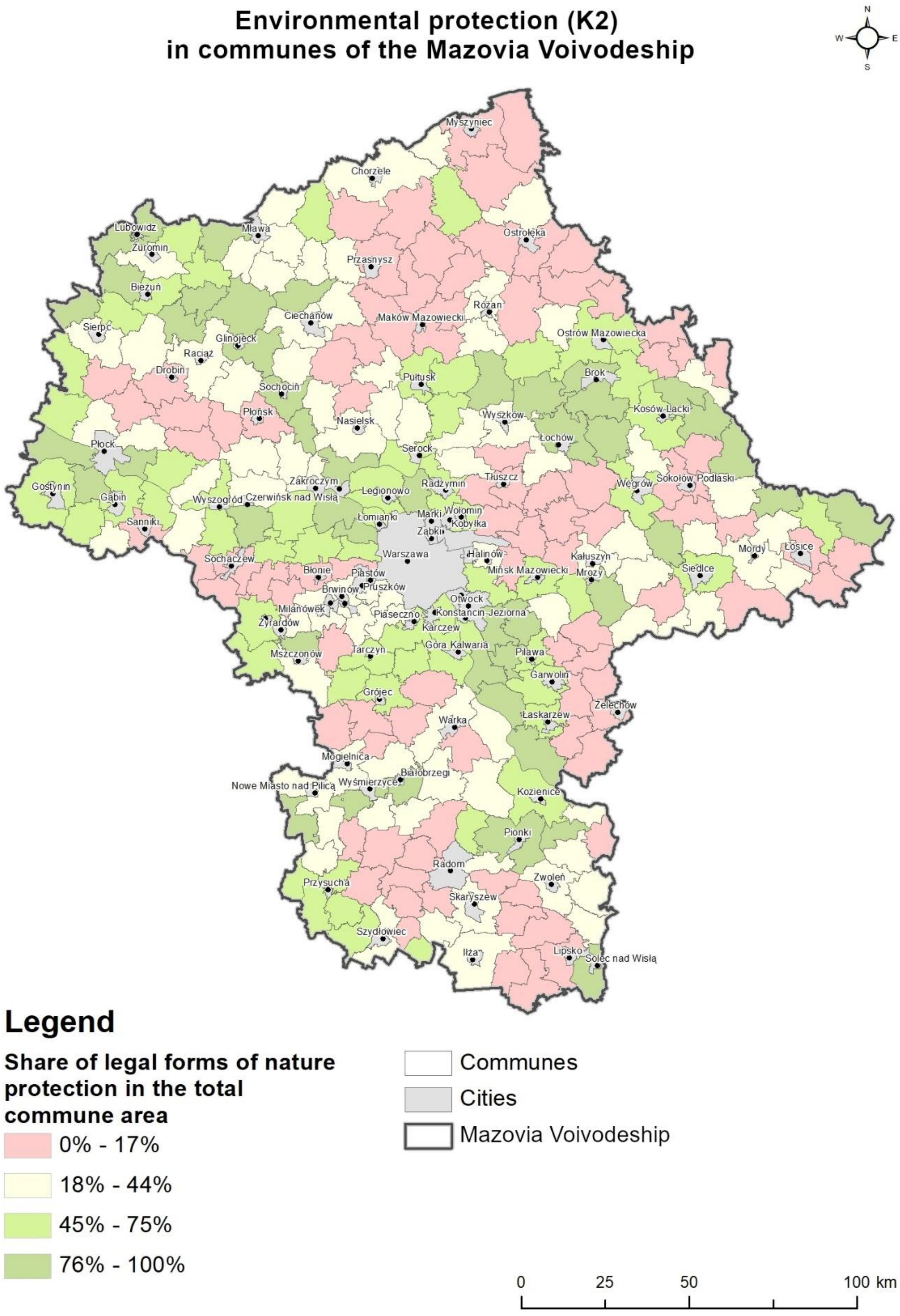

Supplementary Figure S11. Air quality (K3) in the communes of the Mazovia Voivodeship (Source: Own elaboration in ESRI ArcMap 10.8 based on data from AIRLY and NRB)

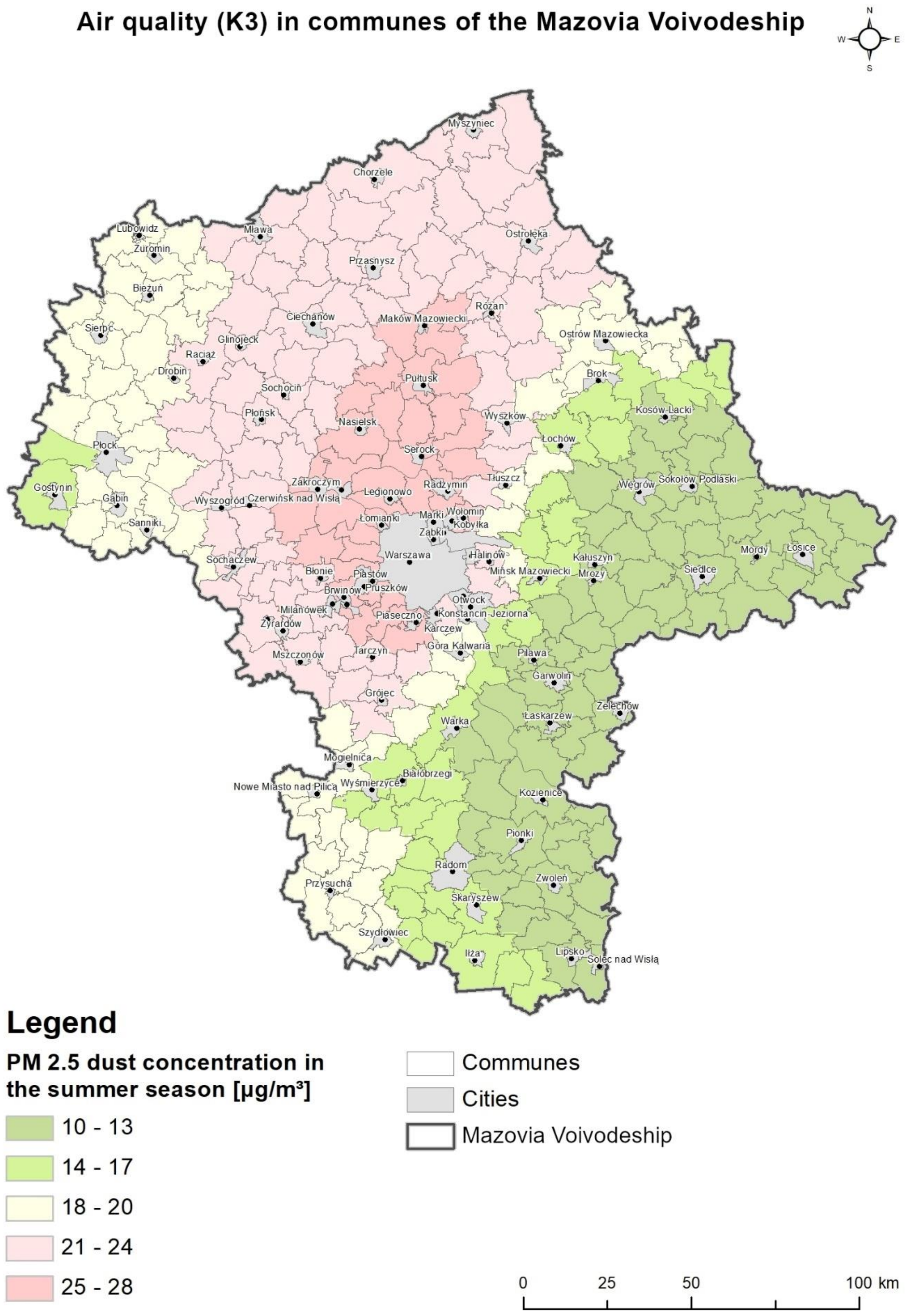

Supplementary Figure S12. Transportation accessibility of communes in the Mazovia Voivodeship (K4) (Source: Own elaboration in ESRI ArcMap 10.8 based on data from OpenStreetMap® and NRB)

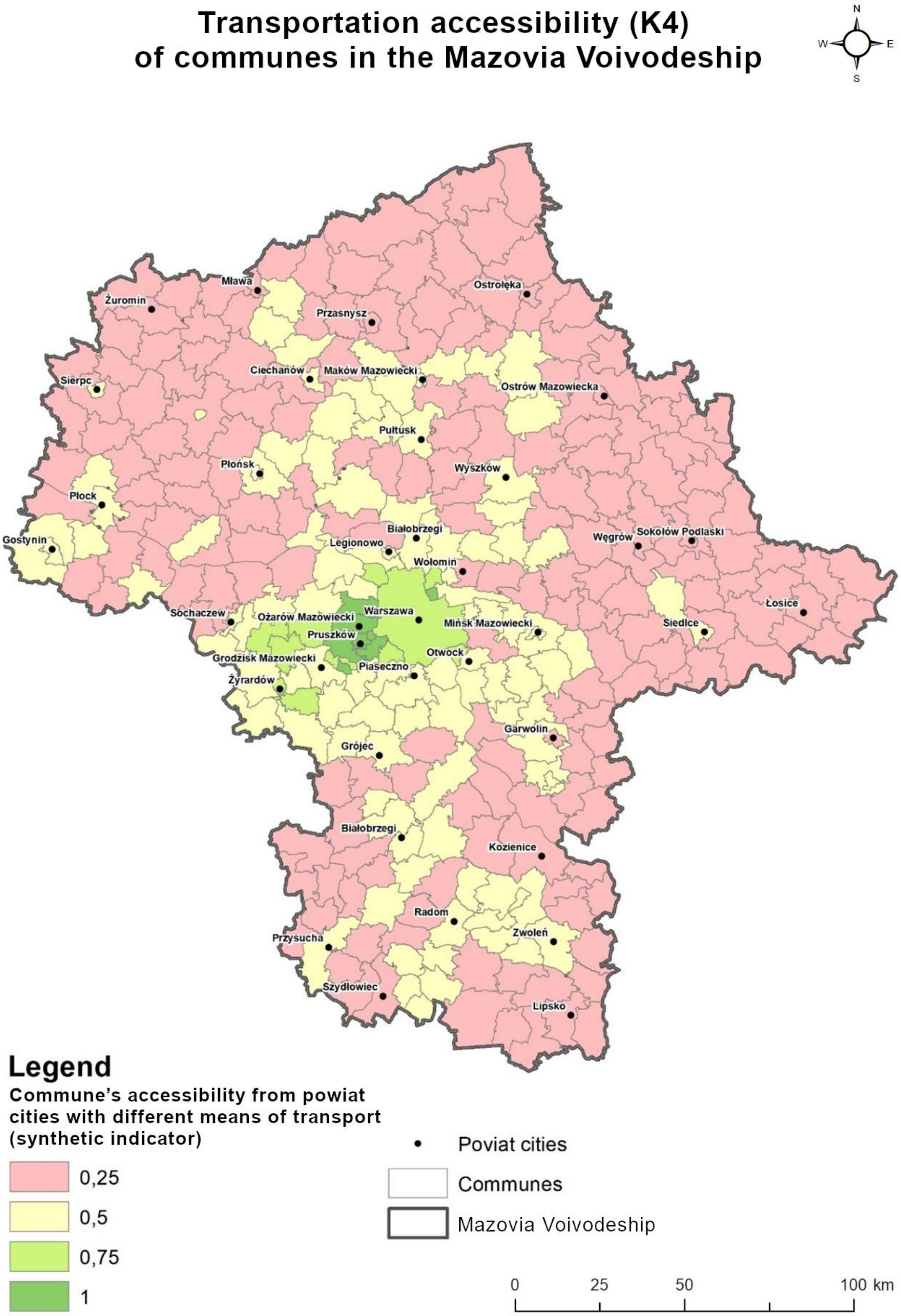

Supplementary Table S5. RPI score of the communes in the Mazovia Voivodeship

No.	Commune's ID	Name	RPI	Recreation potential
1	1414043	Nasielsk	0.1464	very low
2	1411062	Płoniawy-Bramura	0.1699	very low
3	1422072	Przasnysz	0.1706	very low
4	1420052	Dzierzążnia	0.1941	very low
5	1432013	Błonie	0.1984	very low
6	1422052	Krasne	0.2030	very low
7	1415032	Czerwin	0.2056	very low
8	1428072	Sochaczew	0.2057	very low
9	1432063	Ożarów Mazowiecki	0.2079	very low
10	1402042	Gołymin-Ośrodek	0.2095	very low
11	1428082	Teresin	0.2225	very low
12	1419142	Staroźreby	0.2242	very low
13	1411102	Szelków	0.2305	very low
14	1402092	Sońsk	0.2325	very low
15	1418032	Lesznowola	0.2385	very low
16	1424032	Pokrzywnica	0.2412	very low
17	1402072	Opinogóra Górna	0.2445	very low
18	1424062	Winnica	0.2462	very low
19	1411022	Czerwonka	0.2473	very low
20	1420092	Płońsk	0.2514	very low
21	1405062	Żabia Wola	0.2604	very low
22	1434113	Tłuszcz	0.2611	very low
23	1415112	Troszyn	0.2660	low
24	1422032	Czernice Borowe	0.2669	low
25	1411032	Karniewo	0.2686	low
26	1421062	Raszyn	0.2690	low
27	1424012	Gzy	0.2705	low
28	1421042	Michałowice	0.2714	low
29	1415102	Rzekuń	0.2735	low
30	1434072	Klembów	0.2739	low
31	1413062	Stupsk	0.2763	low
32	1425122	Wolanów	0.2778	low
33	1425132	Zakrzew	0.2790	low
34	1419102	Radzanowo	0.2862	low
35	1415083	Myszyniec	0.2865	low
36	1435042	Somianka	0.2886	low
37	1411052	Młynarze	0.2893	low
38	1424052	Świercze	0.2899	low
39	1402052	Grudusk	0.2905	low
40	1420072	Naruszewo	0.2922	low
41	1420102	Raciąż	0.2926	low
42	1402022	Ciechanów	0.2933	low

43	1421033	Brwinów	0.2935	low
44	1416082	Stary Lubotyń	0.2959	low
45	1428052	Nowa Sucha	0.2982	low
46	1416022	Andrzejewo	0.2983	low
47	1428062	Rybno	0.2990	low
48	1411042	Krasnosielc	0.2998	low
49	1411073	Różan	0.3030	low
50	1405043	Grodzisk Mazowiecki	0.3043	low
51	1419012	Bielsk	0.3055	low
52	1406022	Błędów	0.3055	low
53	1414063	Zakroczym	0.3058	low
54	1419053	Drobin	0.3060	low
55	1415072	Łyse	0.3061	low
56	1406032	Chynów	0.3065	low
57	1416092	Szulborze Wielkie	0.3078	low
58	1412052	Dębe Wielkie	0.3080	low
59	1413092	Wieczfnia Kościelna	0.3086	low
60	1410012	Huszlew	0.3095	low
61	1421052	Nadarzyn	0.3099	low
62	1425052	Jedlińsk	0.3108	low
63	1427022	Gozdowo	0.3117	low
64	1413082	Szydłowo	0.3127	low
65	1409033	Lipsko	0.3128	low
66	1411092	Sypniewo	0.3134	low
67	1406062	Jasieniec	0.3142	low
68	1413102	Wiśniewo	0.3178	low
69	1416032	Boguty-Pianki	0.3187	low
70	1420122	Załuski	0.3194	low
71	1420032	Baboszewo	0.3208	low
72	1410023	Łosice	0.3242	low
73	1406012	Belsk Duży	0.3271	low
74	1420082	Nowe Miasto	0.3336	low
75	1424043	Pułtusk	0.3352	low
76	1434052	Dąbrówka	0.3354	low
77	1415042	Goworowo	0.3361	low
78	1437063	Żuromin	0.3376	low
79	1432042	Leszno	0.3382	low
80	1434123	Wołomin	0.3391	low
81	1402062	Ojrzeń	0.3412	low
82	1419042	Bulkowo	0.3423	low
83	1412142	Stanisławów	0.3447	low
84	1418063	Tarczyn	0.3456	low
85	1425072	Kowala	0.3492	low
86	1406083	Nowe Miasto nad Pilicą	0.3498	low
87	1429072	Sabnie	0.3553	low

88	1422062	Krzynowłoga Mała	0.3575	low
89	1429082	Sokołów Podlaski	0.3591	moderate
90	1419132	Stara Biała	0.3612	moderate
91	1406053	Grójec	0.3632	moderate
92	1410062	Stara Kornica	0.3647	moderate
93	1427052	Sierpc	0.3648	moderate
94	1406073	Mogielnica	0.3676	moderate
95	1418052	Prażmów	0.3680	moderate
96	1418043	Piaseczno	0.3684	moderate
97	1415092	Olszewo-Borki	0.3698	moderate
98	1414052	Pomiechówek	0.3704	moderate
99	1438023	Mszczonów	0.3712	moderate
100	1405032	Baranów	0.3714	moderate
101	1422023	Chorzele	0.3728	moderate
102	1412082	Jakubów	0.3758	moderate
103	1408043	Serock	0.3763	moderate
104	1412062	Dobre	0.3765	moderate
105	1416112	Zaręby Kościelne	0.3784	moderate
106	1415052	Kadzidło	0.3793	moderate
107	1437022	Kuczbork-Osada	0.3815	moderate
108	1429062	Repki	0.3821	moderate
109	1423052	Potworów	0.3825	moderate
110	1435062	Zabrodzie	0.3829	moderate
111	1403112	Sobolew	0.3830	moderate
112	1415062	Lelis	0.3833	moderate
113	1425112	Wierzbica	0.3848	moderate
114	1422042	Jednorożec	0.3851	moderate
115	1404032	Pacyna	0.3881	moderate
116	1433022	Grębków	0.3895	moderate
117	1432072	Stare Babice	0.3903	moderate
118	1430022	Jastrząb	0.3917	moderate
119	1412073	Halinów	0.3945	moderate
120	1408052	Wieliszew	0.3964	moderate
121	1427072	Zawidz	0.3966	moderate
122	1406042	Goszczyn	0.3972	moderate
123	1426132	Zbuczyn	0.3981	moderate
124	1413072	Szreńsk	0.3995	moderate
125	1433092	Wierzbno	0.4005	moderate
126	1415022	Czarnia	0.4015	moderate
127	1403143	Żelechów	0.4038	moderate
128	1432022	Izabelin	0.4049	moderate
129	1402082	Regimin	0.4066	moderate
130	1418023	Konstancin-Jeziorna	0.4069	moderate
131	1426062	Paprotnia	0.4086	moderate
132	1409052	Sienno	0.4114	moderate

133	1411082	Rzewnie	0.4118	moderate
134	1409042	Rzeczniów	0.4126	moderate
135	1403082	Miastków Kościelny	0.4138	moderate
136	1425103	Skaryszew	0.4146	moderate
137	1404043	Sanniki	0.4151	moderate
138	1430053	Szydłowiec	0.4158	moderate
139	1425092	Przytyk	0.4172	moderate
140	1408022	Jabłonna	0.4183	moderate
141	1420113	Sochocin	0.4187	moderate
142	1416102	Wąsewo	0.4188	moderate
143	1410032	Olszanka	0.4205	moderate
144	1434082	Poświętne	0.4219	moderate
145	1432053	Łomianki	0.4222	moderate
146	1405052	Jaktorów	0.4229	moderate
147	1404052	Szczawin Kościelny	0.4241	moderate
148	1437013	Bieżuń	0.4253	moderate
149	1434093	Radzymin	0.4260	moderate
150	1436053	Zwoleń	0.4290	moderate
151	1423032	Klwów	0.4323	moderate
152	1435053	Wyszków	0.4332	moderate
153	1416062	Nur	0.4337	moderate
154	1426072	Przesmyki	0.4362	moderate
155	1425062	Jedlnia-Letnisko	0.4365	moderate
156	1425022	Gózd	0.4371	moderate
157	1406113	Warka	0.4379	moderate
158	1438053	Wiskitki	0.4384	moderate
159	1427042	Rościszewo	0.4390	moderate
160	1420062	Joniec	0.4405	moderate
161	1429022	Bielany	0.4415	moderate
162	1419122	Słupno	0.4421	moderate
163	1419082	Mała Wieś	0.4436	moderate
164	1420043	Czerwińsk nad Wisłą	0.4440	moderate
165	1428032	Iłów	0.4451	moderate
166	1409022	Ciepielów	0.4462	moderate
167	1417082	Wiązowna	0.4508	moderate
168	1436012	Kazanów	0.4524	moderate
169	1412112	Mińsk Mazowiecki	0.4540	moderate
170	1430042	Orońsko	0.4545	moderate
171	1417043	Karczew	0.4559	moderate
172	1423082	Wieniawa	0.4569	moderate
173	1419153	Wyszogród	0.4574	moderate
174	1406092	Pniewy	0.4589	moderate
175	1428042	Młodzieszyn	0.4610	high
176	1426082	Siedlce	0.4627	high
177	1426102	Suchożebry	0.4645	high

178	1418013	Góra Kalwaria	0.4652	high
179	1433062	Miedzna	0.4673	high
180	1424072	Zatory	0.4673	high
181	1424022	Obryte	0.4682	high
182	1425033	Iłża	0.4695	high
183	1434062	Jadów	0.4700	high
184	1412093	Kałuszyn	0.4703	high
185	1427032	Mochowo	0.4716	high
186	1433032	Korytnica	0.4716	high
187	1429042	Jabłonna Lacka	0.4719	high
188	1407042	Grabów nad Pilicą	0.4730	high
189	1419022	Bodzanów	0.4730	high
190	1403032	Borowie	0.4756	high
191	1413042	Radzanów	0.4764	high
192	1401022	Promna	0.4770	high
193	1415012	Baranowo	0.4782	high
194	1412132	Siennica	0.4795	high
195	1413052	Strzegowo	0.4822	high
196	1436042	Tczów	0.4824	high
197	1434102	Strachówka	0.4850	high
198	1432032	Kampinos	0.4863	high
199	1419032	Brudzeń Duży	0.4875	high
200	1438042	Radziejowice	0.4884	high
201	1423072	Rusinów	0.4889	high
202	1408032	Nieporęt	0.4894	high
203	1403122	Trojanów	0.4906	high
204	1426053	Mordy	0.4922	high
205	1419063	Gąbin	0.4935	high
206	1413022	Dzierzgowo	0.4947	high
207	1403092	Parysów	0.5039	high
208	1438032	Puszcza Mariańska	0.5045	high
209	1436032	Przyłęk	0.5058	high
210	1426092	Skórzec	0.5083	high
211	1401032	Radzanów	0.5138	high
212	1416072	Ostrów Mazowiecka	0.5162	high
213	1436022	Policzna	0.5191	high
214	1416052	Małkinia Górna	0.5289	high
215	1401042	Stara Błotnica	0.5309	high
216	1402033	Glinojeck	0.5372	high
217	1407072	Sieciechów	0.5383	high
218	1423063	Przysucha	0.5414	high
219	1429092	Sterdyń	0.5429	high
220	1407022	Głowaczów	0.5432	high
221	1403052	Górzno	0.5432	high
222	1407062	Magnuszew	0.5491	high

223	1433042	Liw	0.5515	high
224	1412102	Latowicz	0.5532	high
225	1430012	Chlewiska	0.5579	high
226	1426032	Kotuń	0.5610	high
227	1407032	Gniewoszów	0.5615	high
228	1414032	Leoncin	0.5620	high
229	1403042	Garwolin	0.5624	high
230	1430032	Mirów	0.5635	high
231	1429053	Kosów Lacki	0.5662	high
232	1414022	Czosnów	0.5676	high
233	1404022	Gostynin	0.5766	high
234	1437033	Lubowidz	0.5773	high
235	1425042	Jastrzębia	0.5791	high
236	1410042	Platerów	0.5803	high
237	1423012	Borkowice	0.5846	very high
238	1423042	Odrzywół	0.5858	very high
239	1423022	Gielniów	0.5879	very high
240	1437052	Siemiątkowo	0.5971	very high
241	1426122	Wodynie	0.5984	very high
242	1417052	Kołbiel	0.6001	very high
243	1419112	Słubice	0.6004	very high
244	1401013	Białobrzegi	0.6013	very high
245	1403103	Pilawa	0.6031	very high
246	1410052	Sarnaki	0.6031	very high
247	1437042	Lutocin	0.6045	very high
248	1409012	Chotcza	0.6055	very high
249	1401052	Stromiec	0.6061	very high
250	1433053	Łochów	0.6096	very high
251	1426012	Domanice	0.6128	very high
252	1426042	Mokobody	0.6140	very high
253	1426112	Wiśniew	0.6152	very high
254	1413032	Lipowiec Kościelny	0.6208	very high
255	1435032	Rząśnik	0.6267	very high
256	1407053	Kozienice	0.6319	very high
257	1433082	Stoczek	0.6326	very high
258	1427062	Szczutowo	0.6363	very high
259	1428022	Brochów	0.6445	very high
260	1412042	Cegłów	0.6468	very high
261	1419092	Nowy Duninów	0.6501	very high
262	1426022	Korczew	0.6577	very high
263	1417072	Sobienie-Jeziory	0.6770	very high
264	1401063	Wyśmierzyce	0.6773	very high
265	1417032	Celestynów	0.6781	very high
266	1412123	Mrozy	0.6795	very high
267	1435022	Długosiodło	0.6882	very high

268	1416043	Brok	0.6891	very high
269	1433072	Sadowne	0.6961	very high
270	1435012	Brańszczyk	0.6974	very high
271	1403062	Łaskarzew	0.7060	very high
272	1419072	Łąck	0.7098	very high
273	1407012	Garbatka-Letnisko	0.7111	very high
274	1429032	Ceranów	0.7319	very high
275	1409063	Solec nad Wisłą	0.7373	very high
276	1417062	Osieck	0.7454	very high
277	1403132	Wilga	0.7498	very high
278	1425082	Pionki	0.7792	very high
279	1403072	Maciejowice	0.8343	very high

References:

1. Morse, J. W., Gladkikh, T. M., Hackenburg, D. M. & Gould, R. K. COVID-19 and human-nature relationships: Vermonters' activities in nature and associated nonmaterial values during the pandemic. *PLOS ONE* **15**, 1–23 (2020).
2. Rice, W. *et al.* Longitudinal changes in the outdoor recreation community's reaction to the COVID-19 pandemic: Final report on a three-phase national survey of outdoor enthusiasts. 1–10 Preprint at https://doi.org/10.31235/osf.io/gnjcy (2020).
3. Lesser, I. A. & Nienhuis, C. P. The Impact of COVID-19 on Physical Activity Behavior and Well-Being of Canadians. *International Journal of Environmental Research and Public Health* **17**, 3899 (2020).
4. O'Connell, T., Howard, R. & Hutson, G. *The Impact of COVID-19 on Outdoor Recreation Participation in Canada Initial Report on a National Study of Outdoor Recreationists*. 1–17 (2020).
5. Miazek, P. Przyczyny zróżnicowania ruchu turystycznego w polskich parkach narodowych. *Tourism* **30**, 73–86 (2020).
6. Uglis, J., Kasprzak, K. & Jęczmyk, A. Zanieczyszczenie powietrza a turystyka. *Pol. Jour. for Sus. Dev.* **23**, 17–24 (2019).
7. Buczek-Kowalik, M. Waloryzacja turystyczna środowiska przyrodniczego wybranych gmin Pogórza Dynowskiego i Przemyskiego. in *Problemy ochrony środowiska przyrodniczego i kulturowego Pogórza Dynowskiego w rozwoju turystyki* (ed. Krupa, J.) 309–321 (Związek Gmin Turystycznych Pogórza Dynowskiego, 2016).
8. Jakiel, M. *Ocena atrakcyjność wizualnej krajobrazu dolinek krakowskich – możliwości zastosowania w planowaniu przestrzennym*. vol. 3 (Instytut Geografii i Gospodarki Przestrzennej UJ, 2015).
9. Duda-Seifert, M. Kryteria oceny atrakcyjności turystycznej obiektów architektury w świetle literatury. *Turystyka Kulturowa* **4/2015**, 1–14 (2015).
10. Fornal-Pieniak, B. & Żarska, B. Methods of landscape evaluation for tourism and recreation. *ASP.FC* **13**, 3–9 (2014).
11. Brzezińska-Wójcik, T. & Świeca, A. Environmental determinants of tourism development in the selected municipalities of the Giełczew Elevation (mid-eastern Poland). *Problemy Ekologii Krajobrazu* **27**, 65–72 (2014).
12. Gierańczyk, W. & Gierańczyk, W. Przestrzenne zróżnicowanie infrastruktury turystycznej w Polsce w świetle typologii obszarów wiejskich według OECD i EUROSTAT. *Barometr Regionalny* **11**, 45–55 (2013).
13. Graja-Zwolińska, S. & Uglis, J. Syntetyczna ocena uwarunkowań rozwoju ekoturystyki w województwie wielkopolskim. in *Potencjał turystyczny. Zagadnienia przestrzenne* (ed. Meyer, B.) 609–620 (Wydawnictwo Naukowe Uniwersytetu Szczecińskiego, 2010).
14. Wyrzykowski, J. Potencjał turystyczny w ujęciu geograficznym. *Zeszyty Naukowe Uniwersytetu Szczecińskiego. Ekonomiczne Problemy Usług* **52**, 33–42 (2010).
15. Kowalczyk, A. ZASTOSOWANIE MODELU PŁATY I KORYTARZE DO WALORYZACJI ŚRODOWISKA PRZYRODNICZEGO DLA POTRZEB REKREACJI. in *Płaty i korytarze jako elementy struktury krajobrazu – możliwości i ograniczenia koncepcji* (ed. Cieszewska, A.) vol. XIV (2004).
16. Zajadacz, A. *Potencjał turystyczny miast na przykładzie wybranych miast Sudetów Zachodnich*. (Bogucki Wydawnictwo Naukowe, 2004).
17. Kistowski, M. Rola i zakres studiów fizycznogeograficznych w procedurze sporządzania opracowań ekofizjograficznych. in *Geograficzne aspekty globalizacji i integracji europejskiej* (eds. Śmigielska, M. & Słodczyk, J.) (Polskie Towarzystwo Geograficzne, Uniwersytet Opolski, 2003).
18. Dedio, T. Atrakcyjność jezior obszaru młodoglacjalnego dla rekreacji (na przykładzie jezior Polski Północno-Zachodniej). *Przegląd Geograficzny* **LXI**, 81–94 (1989).
19. Deller, S. & Lledo, V. Amenities and Rural Appalachia Economic Growth. *Agric. resour. econ. rev.* **36**, 107–132 (2007).
20. Ganning, J. P. & Flint, C. G. Constructing a Community-Level Amenity Index. *Society & Natural Resources* **23**, 1253–1258 (2010).
